# Genome-Wide Analysis of the *Catharanthus roseus* RLK1-Like in Soybean and *GmCrRLK1L20* Responds to Drought and Salt Stresses

**DOI:** 10.3389/fpls.2021.614909

**Published:** 2021-03-18

**Authors:** Zhi-Qi Wang, Tai-Fei Yu, Guo-Zhong Sun, Jia-Cheng Zheng, Jun Chen, Yong-Bin Zhou, Ming Chen, You-Zhi Ma, Wen-Liang Wei, Zhao-Shi Xu

**Affiliations:** ^1^College of Agriculture, Yangtze University, Hubei Collaborative Innovation Center for Grain Industry, Engineering Research Center of Ecology and Agricultural Use of Wetland, Ministry of Education, Jingzhou, China; ^2^Institute of Crop Science, Chinese Academy of Agricultural Sciences(CAAS)/National Key Facility for Crop Gene Resources and Genetic Improvement, Key Laboratory of Biology and Genetic Improvement of Triticeae Crops, Ministry of Agriculture, Beijing, China; ^3^College of Agronomy, Anhui Science and Technology University, Fengyang, China

**Keywords:** CrRLK1L, genome-wide analysis, drought, salt, soybean

## Abstract

Abiotic stresses, such as drought and salinity, severely affects the growth, development and productivity of the plants. The *Catharanthus roseus* RLK1-like (CrRLK1L) protein kinase family is involved in several processes in the plant life cycle. However, there have been few studies addressing the functions of CrRLK1L proteins in soybean. In this study, 38 *CrRLK1L* genes were identified in the soybean genome (*Glycine max* Wm82.a2.v1). Phylogenetic analysis demonstrated that soybean *CrRLK1L* genes were grouped into clusters, cluster I, II, III. The chromosomal mapping demonstrated that 38 *CrRLK1L* genes were located in 14 of 20 soybean chromosomes. None were discovered on chromosomes 1, 4, 6, 7, 11, and 14. Gene structure analysis indicated that 73.6% soybean *CrRLK1L* genes were characterized by a lack of introns.15.7% soybean *CrRLK1L* genes only had one intron and 10.5% soybean *CrRLK1L* genes had more than one intron. Five genes were obtained from soybean drought- and salt-induced transcriptome databases and were found to be highly up-regulated. *GmCrRLK1L20* was notably up-regulated under drought and salinity stresses, and was therefore studied further. Subcellular localization analysis revealed that the *GmCrRLK1L20* protein was located in the cell membrane. The overexpression of the *GmCrRLK1L20* gene in soybean hairy roots improved both drought tolerance and salt stresses and enhanced the expression of the stress-responsive genes *GmMYB84*, *GmWRKY40*, *GmDREB-like*, *GmGST15*, *GmNAC29*, and *GmbZIP78*. These results indicated that *GmCrRLK1L20* could play a vital role in defending against drought and salinity stresses in soybean.

## Introduction

Plants are sessile organisms that are subjected to both abiotic and biotic stressors during its life cycle. In order to adapt to adverse environmental factors, plants have evolved several complex signal transduction pathways and signaling mechanisms to defend against cellular damage (Ramachandra Reddy et al., 2004; Mittler, 2006). Some proteins can transmit stresses signals and regulate the expression of stresses-responsive genes, such as protein kinases, transcription factors, and protein phosphatases (Edwards et al., 2000; Shinozaki and Yamaguchi-Shinozaki, 2007; Hundertmark and Hincha, 2008). Of these proteins, protein kinases are a vital regulator that attaches to different ligands, resulting in downstream gene expression (Chinnusamy et al., 2004).

Receptor-like protein kinases (RLKs) were first identified in maize and are similar to animal receptor protein kinases in both their structure and function (Walker and Zhang, 1990). To date, approximately 600 and 1,100 RLKs have been discovered in *Arabidopsis* and rice, respectively (Shiu et al., 2004). RLKs consist of a protein kinase catalytic domain (PKC), a transmembrane domain (TM), and an extracellular ligand-binding domain (ECLB; Walker, 1994). The PKC is an intracellular domain and can promote or repress the expression of downstream genes by phosphorylation and dephosphorylation. The TM consists of 22–28 amino acids (aa) and transmits signals from extracellular to intracellular domains. The ECLBs identify and combine different signal molecules. However, most RLKs possess the N-terminal signal peptide (SP) in the ECLB, which is separate from epidermal growth factor-like repeats (EGFs). Based on differences in the ECLB, RLKs are mainly divided into six categories: LRR-RLKs, S-RLKs, WAK-RLKs, PR5-RLKs, CR4-RLKs, and Lectin-RLKs (Liu et al., 2017). Several studies have demonstrated that RLKs participate in almost all stages of a plant’s life cycle, including growth, development, and stresses response (Wang et al., 2008; Boisson-Dernier et al., 2011). For example, pea *LecRLK* (*PsLecRLK*) is a lectin receptor-like kinases (*LecRLKs*) located in the plasma membrane. Plants with *PsLecRLK*-overexpression displayed enhanced salt stresses tolerance (Vaid et al., 2015). FON1, a member of the LRR-RLK subfamily, can be induced by both drought and ABA treatment (Feng et al., 2014). CRLK1, a calcium-regulated RLK, responds to cold tolerance in plants (Yang et al., 2010).

*Catharanthus roseus* Receptor-Like Kinase 1 Like (CrRLK1L), a subfamily of RLKs, was first identified in *Catharanthus roseus* cell cultures (Schulze-Muth et al., 1996). CrRLK1L has one or two carbohydrate-binding malectin-like domains when compared to other RLKs in ECLBs (Boisson-Dernier et al., 2011; Lindner et al., 2012). Subsequently, CrRLK1L has been found in some plants. For example, 16, 17, and 40 members have been identified in rice, *Arabidopsis*, and cotton, respectively (Acharya et al., 2007; Nguyen et al., 2015; Niu et al., 2016). Several studies have demonstrated that CrRLK1L regulates protein kinase activity by intramolecular phosphorylation (Vaid et al., 2015). Some members of the CrRLK1L family play crucial roles in various kinds of cells (Lindner et al., 2012). For example, ANX1 (ANXUR1) and ANX2 (ANXUR2) are involved in pollen tube growth and development (Boisson-Dernier et al., 2009, 2013; Miyazaki et al., 2009). THE1 (THESEUS1) and HERK1 (HERCULES1) can regulate cell growth in hypocotyls and leaves (Hematy et al., 2007; Hematy and Hofte, 2008; Guo et al., 2009). Meanwhile, FERONIA (FER) is a member of the CrRLK1L subfamily and was first isolated from a pollen tube mutant (Huck et al., 2003). Previous studies demonstrated that FER responded to different hormone signals that contained auxin-mediated root hair growth in *Arabidopsis* (Duan et al., 2010), ethylene, and brassinosteroid (BR)-promoted hypocotyl elongation (Deslauriers and Larsen, 2010; Mao et al., 2015), and ABA-regulated abiotic stresses responses (Chen et al., 2016). Additionally, FER acts as a receptor for the Rapid Alkalization Factor (RALF) peptide ligand that causes a rapid increase in cytoplasmic calcium and inhibits cell elongation in plants (Haruta et al., 2014). Several studies demonstrated that the RALF-FER pathway leads to increases in NADPH oxidase-dependent ROS (reactive oxygen species), which regulates both cell expansion and stresses responses (Thynne et al., 2017). Based on prior research, we speculate that CrRLK1L family proteins are involved in signal transduction, and regulate cell wall integrity in different tissues.

Soybean (*Glycine max* L.) is a drought- and salt-tolerant dicotyledonous plant, although its growth and yield can both be severely affected by drought and salt stresses. There is little published information on the *CrRLK1L* gene family and how it relates to abiotic stresses mechanisms in soybeans. In this study, we identified 38 possible *CrRLK1L* genes in soybean and conducted analyses of their bioinformatics, including phylogenetic relationships, chromosomal location, intron–exon structure, tissue-specific expression patterns, and stresses-related *cis*-elements. Based on RNA-Seq and Quantitative Real-Time Polymerase Chain Reaction (qRT-PCR) methods, we further investigated *GmCrRLK1L20* and whether it was significantly up-regulated when subjected to drought and salt stresses. Our results demonstrated that *GmCrRLK1L20* overexpression enhanced tolerance to drought and salt stresses in soybeans. These findings provide an insight into the foundation of the *GmCrRLK1L20* gene and how it functions in abiotic stresses responses.

## Materials and Methods

### Plant Materials, Growth Conditions, and Stress Treatments

The soybean Williams 82 was used for the experiments conducted in this study. Seedlings were grown in pots with a humus: vermiculite ratio of 1:1 in a growth chamber with about 60% relative humidity, 28°C day/20°C night temperatures, and 14 h light/10 h dark photoperiod 16-day-old seedling were used for drought and salinity treatments. For drought treatment, the soybean seedlings at the four-leaf stage were removed from the soil and placed on filter paper for drought treatment. For salinity treatment, the soybean seedlings roots at the four-leaf stage were immersed in 200 mM NaCl solution. The samples were collected at different times of post-stress exposure for further research.

### Identification of *CrRLK1L* Gene Family in Soybean

The sequences of previously identified *CrRLK1L* genes in *Arabidopsis* and rice were acquired from the TAIR database^1^ and the RGAP database (http://rice.plantbiology.msu.edu), respectively. We utilized BLASTP searches (*E*-value ≤ 1E-5), using the *Arabidopsis* CrRLK1L proteins as queries, to find the soybean *CrRLK1L* gene in the phytozome database^2^. Each candidate GmCrRLK1L protein sequence was then submitted to the online SMART tool^3^ to corroborate the presence of the complete *CrRLK1L* domains. The physicochemical parameters of the soybean *GmCrRLK1L* gene were obtained using the online ExPASyProtParam program^4^.

### Multiple Sequence Alignments and Phylogenetic Tree Construction

The full-length aa sequences of *CrRLK1L* genes obtained for rice, *Arabidopsis*, and the newly identified soybean *GmCrRLK1L* gene were aligned using the ClustalX software with default parameters. MEGA7.0 software was used to construct an unrooted phylogenetic tree using the neighbor-joining method according to the following parameters: pairwise deletion, Poisson model, and 1,000 bootstrap replications.

### Chromosomal Localization Analysis and Structural Characterization

The locations of the *CrRLK1L* genes on soybean chromosomes were obtained from the chromosomal loci in the Phytozome database. The exon-intron organization of soybean *CrRLK1L* genes were analyzed via coding sequences with corresponding full-length sequences using the online program Gene Structure Display Server (GSDS)^5^. The conserved motifs of the identified soybean CrRLK1L protein sequences were determined using the MEME online program^6^.

### Expression Pattern Analysis of *GmCrRLK1L* Genes in Different Tissues

Transcriptome data were retrieved from the Phytozome database to analyze the expression patterns of *GmCrRLK1L* genes in different tissues, including seeds, roots, root hairs, stem, leaves, flowers, and nodules. The heatmap was constructed using the HemI software.

### Analysis of *Cis*-Acting Elements in *GmCrRLK1L* Gene Promoters

The 2.0 kb region of 5′ UTR upstream of the soybean *CrRLK1L* genes obtained from the Phytozome database were submitted to PlantCARE^7^ to identify ten *cis*-elements, including ABA-responsive elements (ABRE), anaerobic induction elements (ARE), low-temperature responsiveness elements (LTR), defense and stress responsiveness elements (TC-rich repeats), wound-responsive elements (WUN-motif), MYB binding sites involved in drought-inducibility (MBS), W-Box, MYB, MYC, and DRE.

### RNA Extraction and qRT-PCR

The plants at the four-leaf stage were grown in a greenhouse (light period: 14 h light/10 h dark, temperatures: 25°C day/20°C night, relative humidity: 60%). They were then taken out of the pot and washed with water. For drought treatment, the soybean seedlings at the four-leaf stage were placed on filter paper, and samples were collected at 0, 1, 2, 4, 8, 12, and 24 h of post-stress exposure. For salt treatment, the soybean seedlings at the four-leaf stage were immersed in 200 mM of NaCl, and samples were also collected at 0, 1, 2, 4, 8, 12, and 24 h of post-stress exposure. The samples were immediately frozen at −80°C in liquid nitrogen. The total RNA was extracted using a Plant Total RNA Extraction Kit according to the manufacturer’s instructions (TIANGEN). The qRT-PCR was conducted according to the method provided by the Prime Script TM RT Kit (Takara, Shiga, Japan). The primers for qRT-PCR of the five soybean *GmCrRLK1L* genes from the *de novo* soybean transcriptome sequencing were designed by Primer Premier 5.0, while the soybean actin gene was used as a control. An ABI Prism 7500 real-time PCR system (Applied Biosystems, Foster City, CA, United States) was used to perform qRT-PCR (Choi et al., 2005). The data were analyzed by the 2^–ΔΔCT^ method (Le et al., 2011).

### Subcellular Localization of GmCrRLK1L20

The full-length cDNA sequence of *GmCrRLK1L20* was cloned into the N-terminus hGFP protein, which was driven by the CaMV35S promoter. The 35S:GFP vector was used as a control. The recombinant plasmid of *GmCrRLK1L20*-GFP was transformed into *Arabidopsis* protoplasts using a PEG4000-mediated method (He et al., 2016). The fluorescence signal was observed using a confocal laser scanning microscope after incubating it in darkness at 22°C for 18–20 h (Zeiss LSM 700, Oberkochen, Germany) (Riechmann et al., 2000).

### Drought and Salt Stresses Assays of Soybean Hairy Root Composite Plants

Transgenic hairy root composite soybean plants were constructed using the method described by Shi et al. (2018) (Shi et al., 2018). Twelve Williams 82 soybeans were placed in each pot. Three pots formed a group. Drought treatment was conducted for 2 weeks. Under salt stresses, transgenic soybeans and EV-Control seedlings were grown in 200 mM of NaCl for four days. Both the drought and salt treatment experiments were conducted a minimum of three times. Both the treated and the untreated soybean hairy roots were washed with water for RNA isolation and physiological and biochemical experiments. The contents of Catalase (CAT), chlorophyll, Peroxidase (POD), Superoxide dismutase (SOD), Proline (Pro), relative electrical conductivity, and Malondialdehyde (MDA) were measured with the corresponding kit produced by Suzhou Comin Biotechnology Co., Ltd. (Suzhou, China) based on the manufacturer’s instructions; all data used were the average of three biological replicates.

### Trypan Blue and NBT Staining

The Williams 82 seedlings with hairy roots approximately 2–5 cm in size were placed in a pot for 5 days, after which they were subjected to either 2 weeks of drought or 200 mM of NaCl for 4 days in a growth chamber. For trypan blue staining, the samples were immersed in a 0.5% Trypan blue (BioDee, China) solution for 12 h and then in 75% ethanol for decoloring until the leaves become white. For NBT staining, the samples were immersed in an NBT staining solution for 12 h and then in 75% ethanol (Wang et al., 2017) until the leaves became white. The pictures were taken with a Canon 50D (Canon, Japan) camera.

### Fresh Weight of Roots

The fresh weight of *GmCrRLK1L20*-RNAi, *GmCrRLK1L20-*OE, and the EV-Control were measured under normal conditions as well as drought and salt conditions. All data used are the average of three biological replicates.

### Statistical Analyses

All experiments were performed twice with at least three independent replicates. The data were assessed with the Student’s *t*-test using functions in Excel 2007. Vertical bars demonstrate ± SE of three biological replicates. An ANOVA test indicated that there were significant differences (^∗^*p* < 0.05, ^∗∗^*p* < 0.01).

### Primers

The primers and sequences used in this study are listed in Supplementary Table 1.

## Results

### Identification of RLK Subfamily *CrRLK1L* Genes in the Soybean Genome

Whole-length proteins and conserved domains of 17 *Arabidopsis CrRLK1L* genes were used as queries to search the soybean genome database. We identified 38 soybean *CrRLK1L* genes in the soybean genome, which were named GmCrRLK1L01-GmCrRLK1L38 based on their chromosomal locations (Table 1). Gene features, including the length of the aa, the protein molecular weight (MW), isoelectric point (p*I*), and the subcellular location were analyzed (Table 1). Of the 38 soybean CrRLK1L proteins, GmCrRLK1L02 was the smallest protein with 647 aa. The largest one was 1186 aa GmCrRLK1L36. The molecular weights (MW) of the proteins varied from 72.72 kDa GmCrRLK1L02 to 133.95 kDa GmCrRLK1L36, and the p*I* ranged from 5.24 GmCrRLK1L03 to 8.81 GmCrRLK1L25. Subcellular localization of each soybean CrRLK1L protein were predicted by the TargetP software of the CBS database (Yu et al., 2004). The predicted subcellular localization results indicated that 38 soybean CrRLK1L proteins were all located in the plasma membrane (PM).

**TABLE 1:** Features of GmCrRLK1L genes identified in soybean.

Number	Gene ID	Gene name	Chromosome	Gene location (bp)	Amino acid (aa)	MW (kDa)	P^I^	Pfam	Localization
1	Glyma.02G121900	GmCrRLk1L01	2	12112034–12115054	820	92.14	8.23	12819/07714	PM
2	Glyma.02G122000	GmCrRLk1L02	2	12115287–12118397	647	72.72	5.81	12819/07714	PM
3	Glyma.03G247800	GmCrRLk1L03	3	44449869–44452478	869	96.22	5.24	12819/07714	PM
4	Glyma.05G099900	GmCrRLk1L04	5	26455876–26459874	793	88.41	6.30	12819/07714	PM
5	Glyma.08G248900	GmCrRLk1L05	8	21678304–21680829	842	92.87	6.25	12819/07714	PM
6	Glyma.08G249200	GmCrRLk1L06	8	21727446–21730345	871	96.61	6.24	12819/07714	PM
7	Glyma.08G249400	GmCrRLk1L07	8	21748017–21750483	790	88.61	6.02	12819/07714	PM
8	Glyma.09G024700	GmCrRI,k1l,08	9	1987676–1991192	852	93.58	5.59	12819/07714	PM
9	Glyma.09G133000	GmCrRI,k1l,09	9	33072536–33075051	818	90.91	7.04	12819/07714	PM
10	Glyma.09G273300	GmCrRRk1R10	9	48955473–48959271	896	98.07	5.64	12819/07714	PM
11	Glyma.10G163200	GmCrRRk1R1 1	10	39716929–39719517	862	95.92	5.67	12819/07714	PM
12	Glyma.10G231500	GmCrRLk1L12	10	46111104–46113584	826	91.81	7.94	12819/07714	PM
13	Glyma.12G074600	GmCrRRk1R1 3	12	5628367–5631944	837	92.74	5.86	12819/07714	PM
14	Glyma.12G148200	GmCrRLk1L14	12	20759752–20763257	846	93.19	5.68	12819/07714	PM
15	Glyma.12G220400	GmCrRLk1L15	12	37978471–37981634	689	75.71	6.44	12819/07714	PM
16	Glyma.12G235900	GmCrRLk1L16	12	39l77716–39l81109	878	96.32	5.77	12819/07714	PM
17	Glyma.13G053600	GrnCrRLk1L17	13	15089069–15092606	894	99.35	5.90	12819/07714	PM
18	Glyma.13G053700	GinCrRI,k1l,18	13	15096852–15099898	819	91.46	5.63	12819/07714	PM
19	Glyma.13G053800	GinCrRl,k1l,19	13	15110071–15112808	702	77.97	6.44	12819/07714	PM
20	Glyma.13G054200	GmCrRLkUGo	13	151ll990–151l8021	787	88.73	8.48	12819/07714	PM
21	Glyma.13G054300	GinCrRI,k1l,21	13	15153615–151561l9	844	94.10	5.98	12819/07714	PM
22	Glyma.13G054400	GinCrRI,k1l,22	13	1516l832–15168321	896	99.53	6.11	12819/07714	PM
23	Glyma.13G201400	GinCrRI,k1l,23	13	31502069–31505627	869	95.29	5.85	12819/07714	PM
24	Glyma.15G042900	GinCrRl,k1l,2-l	15	3373579–3379318	741	81.92	7.93	12819/07714	PM
25	Glyma.16G179600	GmCrRLk1L25	16	34010502–34013669	773	86.06	8.81	12819/07714	PM
26	Glyma.17G102600	GinCrRI,k1l,26	17	803l007–8036592	861	95.69	6.55	12819/07714	PM
27	Glyma.17G166200	GnCrRLk1L27	17	15165279–15168381	843	93.93	5.80	12819/07714	PM
28	Glyma.18G215800	GinCrRI,k1l,28	18	50287921–50291401	894	97.76	5.66	12819/07714	PM
29	Glyma.18G269900	GmCrRLk1L29	18	55344388–55346997	869	97.23	6.26	12819/07714	PM
30	Glyma.18G270100	GinCrRLk1L30	18	55367099–55370356	868	97.39	6.09	12819/07714	PM
31	Glyma.18G270600	GmCrRLk1L31	18	55411917–55419846	1123	124.55	5.77	12819/07714	PM
32	Glyma.18G270700	GinCrRl,k1l,32	18	55425250–55428334	857	95.77	6.25	12819/07714	PM
33	Glyma.18G270900	GinCrRl,k1l,33	18	55438919–55441546	875	97.45	5.82	12819/07714	PM
34	Glyma.18G271000	GnCrRLk1L34	18	55447085–55449676	863	96.85	5.90	12819/07714	PM
35	Glyma.18G271100	GnCrRLk1L35	18	55455768–55458641	883	98.04	6.02	12819/07714	PM
36	Glyma.19G033100	GinCrRI,k1l,36	19	4270496–4278256	1186	133.95	6.49	12819/07714	PM
37	Glyma.20G162300	GnCrRLk1L37	20	40007433–40010671	840	93.18	8.17	12819/07714	PM
38	Glyma.20G225800	GinCrRl,k1l,38	20	45989681–45993317	843	93.73	5.80	12819/07714	PM

### Phylogenetic Analysis of the *CrRLK1L* Family in *Arabidopsis*, Rice, and Soybean

**FIGURE 1:**
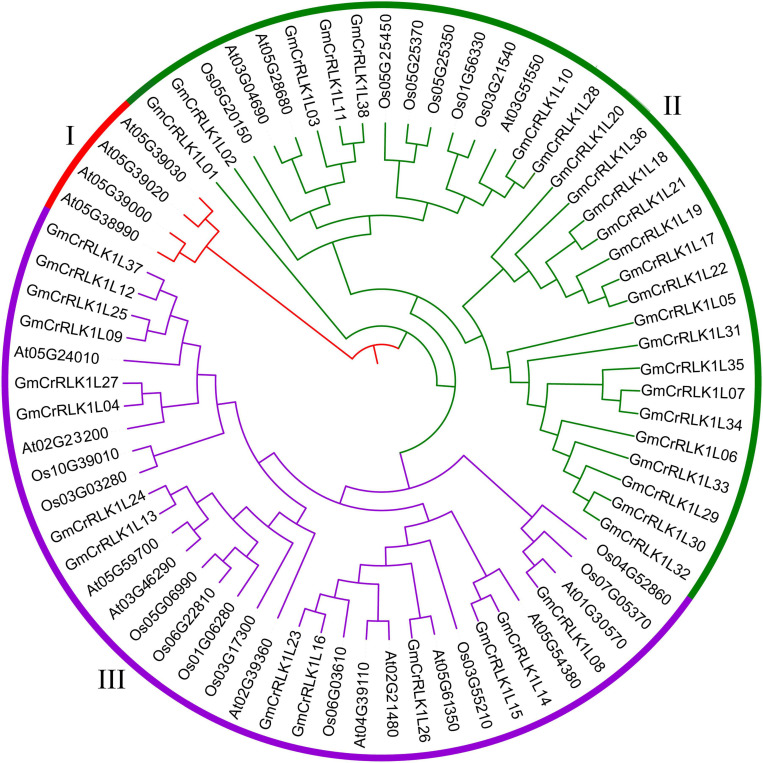
Phylogenetic tree analysis of *CrRLK1L* genes. The full-length amino acid sequence of the CrRLK1L protein from *Arabidopsis*, rice, and soybean were aligned by Crustal W and constructed by the NJ (Neighbor-joining) method with 1,000 bootstrap replicates. Distinct subfamilies are marked by different colors.

To investigate the evolutionary relationship among the *CrRLK1L* genes in soybean, *Arabidopsis*, and rice, an unrooted phylogenetic tree was constructed by aligning the full-length aa sequences from 38 soybean members, 17 *Arabidopsis* members, and 16 rice members. As shown in Figure 1, the 71 *CrRLK1L* genes were divided into three main groups. The soybean *CrRLK1L* family had 24 members in cluster II and 14 members in cluster III. The *Arabidopsis CrRLK1L* had 4 members in cluster I, 3 in cluster II, and 10 in cluster III. The rice *CrRLK1L* had 6 members in cluster II and 10 members in cluster III.

### *GmCrRLK1L* Gene Distribution on Soybean Chromosome

**FIGURE 2:**
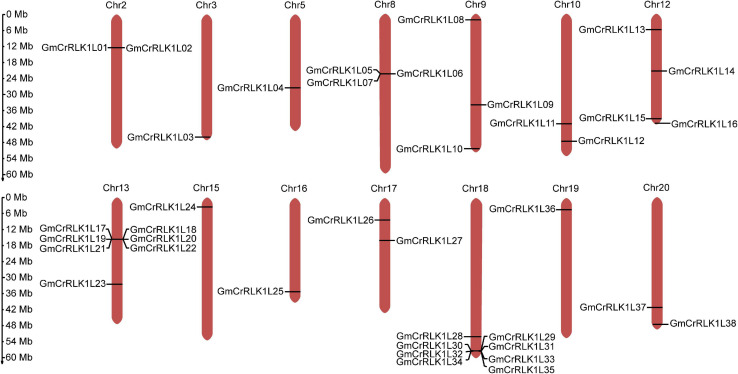
Chromosome distribution of the 38 *GmCrRLK1L* genes. Chromosome location map drawn with MG2C software. *GmCrRLK1L* genes distribution on 14 soybean chromosomes.

Detailed information on the location of the 38 soybean *CrRLK1L* genes used in this study was obtained from Phytozome (Table 1). As shown in Figure 2, 38 CrRLK1L family members are distributed on 14 of 20 soybean chromosomes, with none found on chromosomes 1, 4, 6, 7, 11, and 14. The highest number of soybean *CrRLK1L* genes are found on chromosome 18, while the fewest number of *GmCrRLK1L* genes are found on chromosomes 3, 5, 8, 15, 16, and 19. Eight soybean *CrRLK1L* members were located on chromosome 18, seven soybean *CrRLK1L* members were located on chromosome 13, four soybean *CrRLK1L* members were located on chromosome 12, two soybean *CrRLK1L* members were located on chromosomes 2, 9, 10, 17, and 20, and one soybean *CrRLK1L* member was located on chromosomes 3, 5, 8, 15, 16, and 19.

### Gene Structure and Motif Composition of Soybean *CrRLK1L* Genes

**FIGURE 3:**
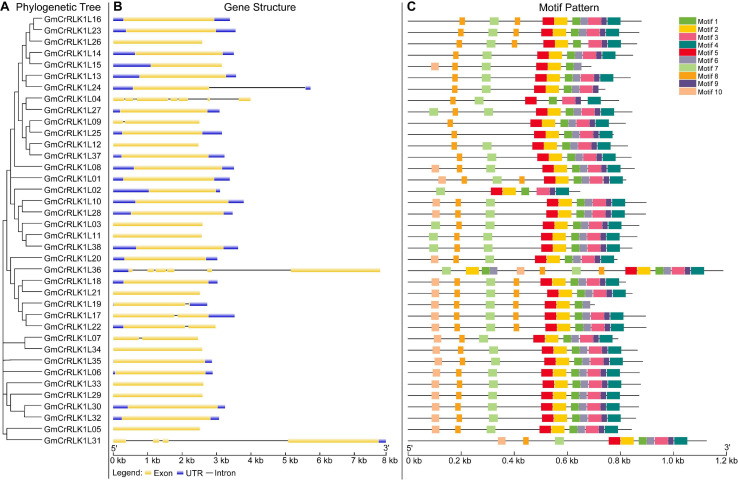
Phylogenetic relationship, gene structure, and architecture of conserved protein motif in CrRLK1L genes from soybean. **(A)** The phylogenetic tree analysis of CrRLK1L genes from soybean. **(B)** Exon/intron organization of *GmCrRLK1L* genes. Yellow boxes represent exons and black lines represent introns. **(C)** The motif composition of soybean CrRLK1L proteins. The motif, numbers 1–10, are displayed in different color boxes. For details of motifs refer to Table 2.

We examined the exon–intron organization of all identified soybean *CrRLK1L* genes to better understand the evolution of the soybean *CrRLK1L* family. As shown in Figure 3B, this included very few introns, which was similar to the previous results conducted by Li et al. (2015). However, we found that several genes contained introns: among soybean *CrRLK1L* genes, more than half (28 soybean *CrRLK1L* genes, 73.6%) were free introns. Six soybean *CrRLK1L* genes (15.7%) had one intron and only four soybean *CrRLK1L* genes (10.5%) had more than one intron: GmCrRLK1L04 (seven introns), GmCrRLK1L36 (five introns), and GmCrRLK1L31 (three introns). The MEME website was used to identify the conserved motifs of soybean *CrRLK1L* genes, and 10 motifs were identified (Figure 3C). The lengths of these conserved motifs ranged from 29 to 50 aa. The 10 putative motifs are displayed in Table 2. Thirty-eight soybean *CrRLK1L* genes contained Motif 1 and Motif 5. Motif 2 and Motif 8 were found in 37 soybean *CrRLK1L* genes, with the exception of GmCrRLK1L04 and GmCrRLK1L02. The majority of soybean CrRLK1L proteins (94.7%) contained Motif 3, Motif 7, and Motif 9. Meanwhile, Motif 4 and Motif 5 were found in 35 soybean *CrRLK1L* genes and Motif 10 was only found in 22 soybean *CrRLK1L* genes. Soybean *CrRLK1L* genes in the same groups were generally found to possess a close motif element. However, most functions of these conserved motifs have yet to be explained.

**TABLE 2:** List of the putative motifs of GmCrRLK1L proteins.

Motif	Width	Best possible match
1	29	SWKQRLZICIGAARGLHYLHTGAKHTIIH
2	50	IKRLKPGSQQGLQEFQTEIEMLSQLRHRHLVSLIGYCNENNEMI LVYDFM
3	50	HVSTAVKGSFGYLDPEYYKRQQLTEKSDVYSFGVVLFEVLCARP PLIPTL
4	50	IVDPTLKGQIAPECLKKFGETAEKCLLEDGTDRPSMGDVLWNL EFALQLQ
5	44	BLCRHFSLAEIKAATNNFDESLIIGVGGFGNVYKGYIDDGSTVV
6	29	RDVKTTNILLDENWVAKVSDFGLSRIGPT
7	34	NGTLNMNFNLTWZFPVDSGFTYLLRLHFCEJDPN
8	29	VSAGPKFLRLFFYPASYQSFDRTKASFSV
9	50	INKPGDRVFFIYIADQLAEBWADVLQWSHNQKGVPVVRNYAVLI PGBNTQ
10	29	LYSIETSFALETVYRJNVGGQEISPSNDT

### Expression Pattern Analysis of *GmCrRLK1L* Genes in Different Tissues

**FIGURE 4:**
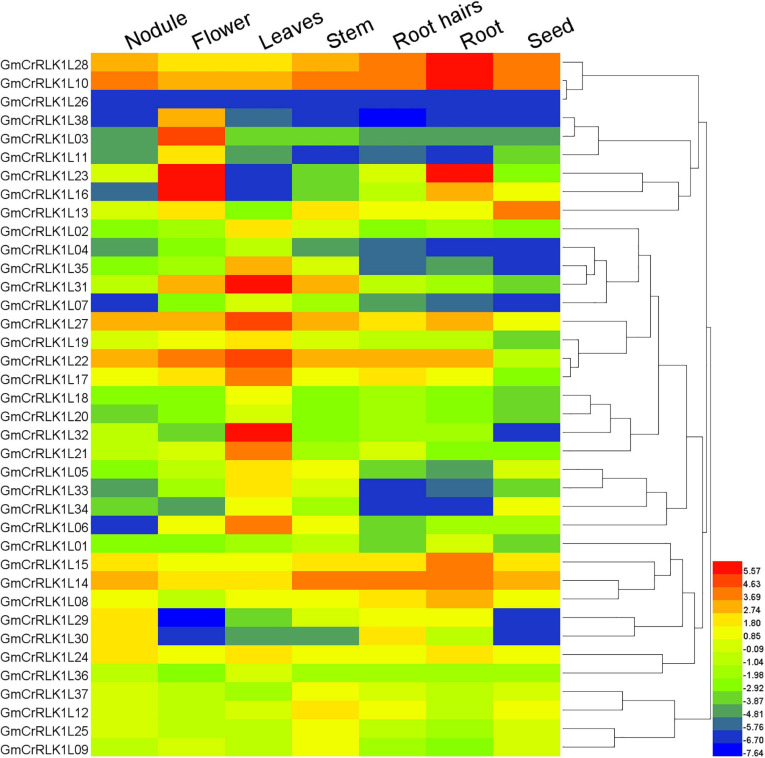
Expression profiles of soybean *CrRLK1L* genes from seven soybean tissues (seeds, roots, root hairs, stem, leaves, flowers, and nodules). Gene expression values are represented by different colors. The color scale is displayed at right of figure.

Different genes possess different expression abundances in different tissues or organs. This helps them adjust their physiological process. Our analyses of 38 soybean *CrRLK1L* gene expression patterns in seven tissues, including seeds, roots, root hairs, stems, leaves, flowers, and nodules, were conducted using known RNA-seq data from soybean genome databases. A heatmap of 38 soybean *CrRLK1L* genes was constructed using the HemI software. As shown in Figure 4, 32 *GmCrRLK1L* genes were expressed in at least one tissue, with the exception of GmCrRLK1L26, GmCrRLK1L04, GmCrRLK1L07, GmCrRLK1L20, GmCrRLK1L01, and GmCrRLK1L36. GmCrRLK1L38, GmCrRLK1L03, and GmCrRLK1L11 were only highly expressed in the flower. Approximately 11 genes (28.9%) were highly expressed in roots and root hairs, demonstrating that the soybean *CrRLK1L* genes could play vital roles in responding to adverse environmental factors.

### Stress-Related *cis*-Elements in Soybean *CrRLK1L* Genes Promoters

**FIGURE 5:**
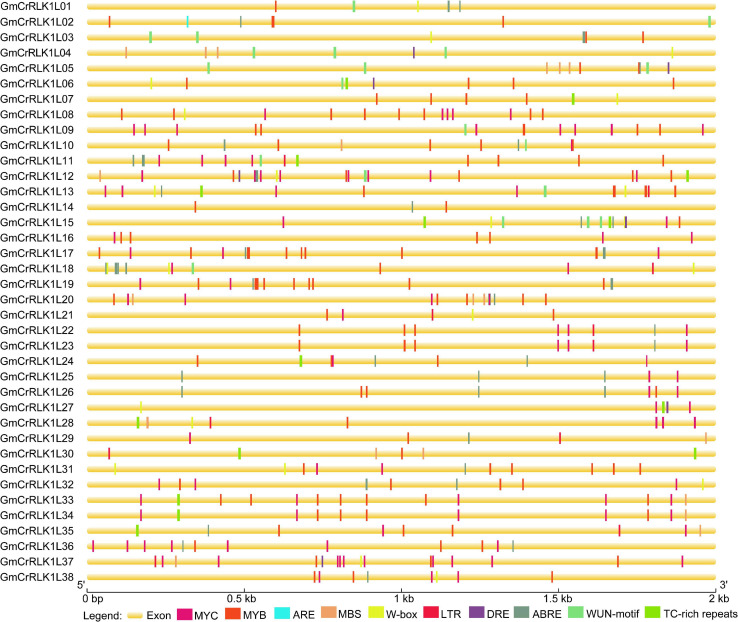
Predicted *cis*-elements in soybean *CrRLK1L* gene promoter’s region. Different *cis*-elements are marked by distinct color blocks and are located in relative positions on the promoter. The ABA-responsive element (ABRE), anaerobic induction element (ARE), low-temperature responsiveness element (LTR), defense and stresses responsiveness element (TC-rich repeats), wound-responsive element (WUN-motif), MYB binding site involved in drought-inducibility (MBS), W-Box, MYB, MYC, and DRE were analyzed. The upstream length to the translation starts site can be obtained using the scale at the bottom.

To further analyze possible regulatory mechanisms of soybean *CrRLK1L* genes among abiotic and biotic stress responses, the 2.0 kb upstream sequences from the start codon ATG of *CrRLK1L* genes were submitted to PlantCARE (see text footnote 7) to detect stresses-related cis-elements. Ten stresses response elements, including ABRE, MYB, MYC, ARE, LTR, W-box, WUN-motif, MBS, DRE, and TC-rich repeats were identified in these 38 soybean *CrRLK1L* gene promoters (Figure 5). Our results demonstrated that 38 soybean *CrRLK1L* genes have at least one cis-element related to stresses response, which demonstrated that soybean *CrRLK1L* gene expression could be associated with stresses response. For example, 36 and 29 soybean *CrRLK1L* genes (94.7%) have one or more MYB and MYC, respectively, and 24 soybean *CrRLK1L* genes possessed the ABA-responsive element. W-box, MBS, and LTR-responsive elements were found in 17, 12, and 11 soybean *CrRLK1L* genes, respectively. WUN-motif and TC-rich repeats were found on 13 soybean *CrRLK1L* genes. Seven DRE and one ARE were found on soybean *CrRLK1L* genes, respectively. The *cis*-elements analysis indicated that soybean *CrRLK1L* genes may be involved in different stresses responses.

### Expression Pattern Analysis of Five Soybean *CrRLK1L* Genes Under Drought and Salt Stresses Conditions

**FIGURE 6:**
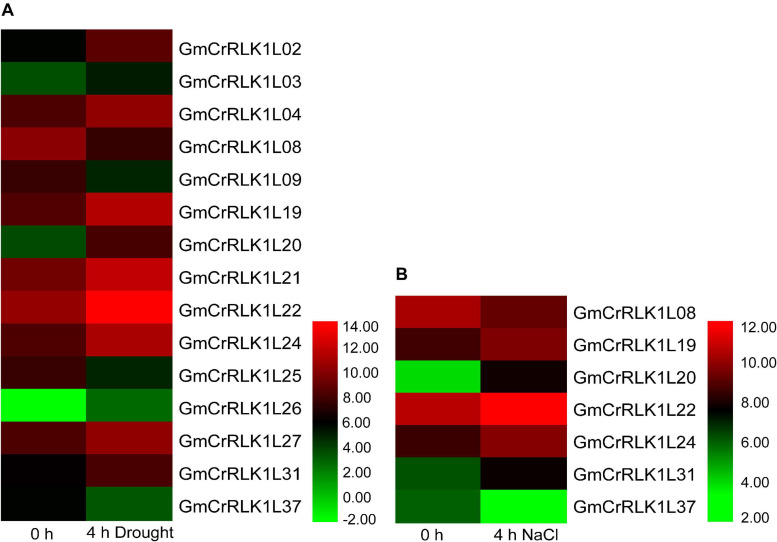
Hierarchical clustering of expression profiles of 15 and 7 drought- and NaCl-responsive genes, respectively. **(A)** The expression level of 15 drought responsive genes. **(B)** The expression level of 7 NaCl responsive genes. The color scale is shown at right of figure.

**FIGURE 7:**
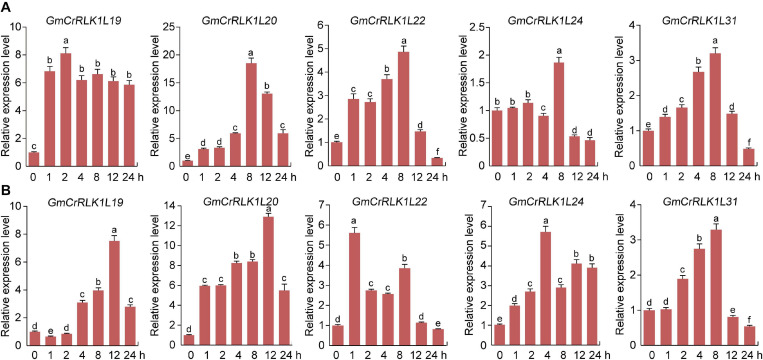
Analysis of expression patterns of five selected *CrRLK1L* genes under drought and salt treatment by qRT-PCR. **(A)** qRT-PCR of five *GmCrRLK1L* genes under drought treatment. **(B)** qRT-PCR of five *GmCrRLK1L* genes under salt treatment. The soybean Actin (U60506) was used as an internal control. The X-axes and Y-axes indicate time and relative expression levels, respectively. The data represent means ± SD of three biological replications.

To thoroughly analyze the transcript levels of *CrRLK1L* genes in soybean under drought and salt stresses, we selected five CrRLK1L family members from the *de novo* transcriptome sequencing of soybean: *GmCrRLK1L19*, *GmCrRLK1L20*, *GmCrRLK1L22*, *GmCrRLK1L24*, and *GmCrRLK1L31*, which was based on the value of log_2_ (GH_treat/CK1_treat) and log_2_ (NaCl_treat/CK2_treat) > 1 (Figure 6). We also performed an expression pattern analysis of 5 *CrRLK1L* genes via qRT-PCR (Figure 7). These five *GmCrRlLK1L* genes were all upregulated under drought and salt stresses conditions (Figure 7), which was consistent with the hierarchical clustering found in expression profiles from drought and NaCl RNA-seq (Figure 6). Under drought treatment, 4 soybean *CrRLK1L* genes, *GmCrRLK1L20, GmCrRLK1L22, GmCrRLK1L24*, and *GmCrRLK1L31*, all peaked at 8 h (peaks of ∼ 20-, 5-, 2-, and 3-fold, observed at same time points, respectively). However, *GmCrRLK1L19* peaked at 2 h (∼8-fold). Meanwhile, under NaCl condition, *GmCrRLK1L22, GmCrRLK1L24*, and *GmCrRLK1L31* peaked at 1 h (∼6-fold), at 4 h (∼6-fold), and at 8 h (∼3-fold), respectively. *GmCrRLK1L19* and *GmCrRLK1L20* were all significantly upregulated (approximately 8- and 12-fold) at 12 h, respectively. By analyzing the levels of the expression pattern of *GmCrRLK1L* genes, we discovered that *GmCrRLK1L20* had the highest expression level after 8 h of drought treatment (∼18-fold) and 12 h of high-salt conditions (∼13-fold). The qRT-PCR results showed that *GmCrRLK1L20* had the highest expression level under drought and salt treatment. As such, *GmCrRLK1L20* was used for further study. The expression data of the five genes were placed in the attachment Supplementary Table 3.

### Subcellular Localization of *GmCrRLK1L20*

**FIGURE 8:**
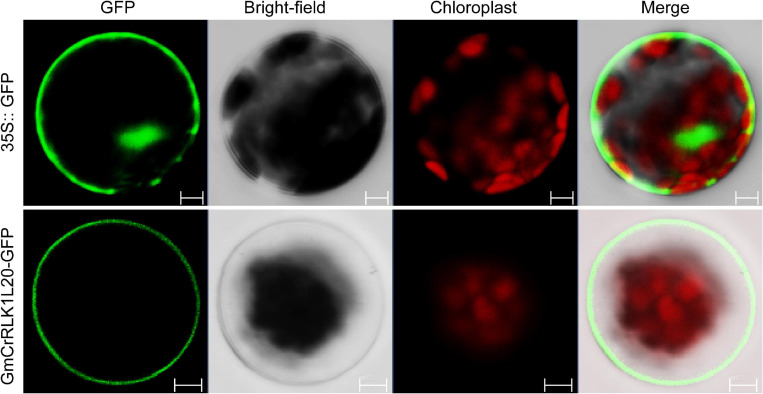
Subcellular Localization analysis of the GmCrRLK1L20 Protein within the cell by GFP assays. 35S:GFP, control with empty vector; GmCrRLK1L20-GFP, GmCrRLK1L20-GFP fusion. Bars = 5 μm.

To analyze the subcellular localization of *GmCrRLK1L20*, the open reading frame (ORF) sequence (without the stop codon of the *GmCrRLK1L20* gene) was fused to the N-terminal of the humanized green fluorescent protein (hGFP) reporter protein and co-transformed into *Arabidopsis* protoplasts to identify the localization of the GFP fluorescence signal. The 35S:GFP vector was used as a control. The fluorescence signal of *GmCrRLK1L20* was specifically detected in the cell membrane using a confocal laser scanning microscope, while the GFP fluorescence signal was identified throughout the whole cell (Figure 8).

### *GmCrRLK1L20* Improved Drought and Salt Tolerance in Transgenic Soybean Hairy Roots

**FIGURE 9:**
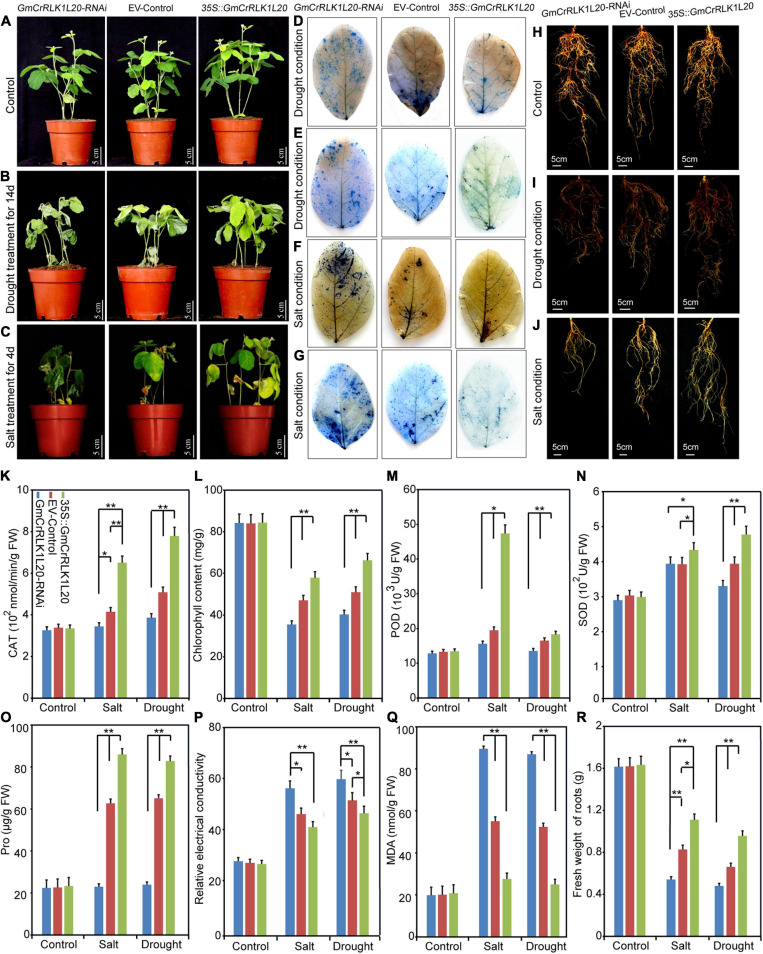
Phenotype and function analysis of soybean GmCrRLK1L20 in transgenic soybean hairy roots under drought and salt stresses conditions. **(A–C)** growth conditions of *GmCrRLK1L20*-RNAi, EV-Control (the empty plasmid of pCAMBIA3301), and *GmCrRLK1L20*-OE under normal, drought, and salt stresses conditions, respectively. NBT **(D,F)** staining of the leaves of GmCrRLK1L20-RNAi, EV-Control, and GmCrRLK1L20-OE under drought and salt conditions, respectively. The brightness of the leaf color indicates H_2_O_2_ and O_2_^–^ contents **(D,F)**. trypan blue **(E,G)** staining of the leaves of GmCrRLK1L20-RNAi, EV-Control, and GmCrRLK1L20-OE under drought and salt stresses conditions, respectively. Trypan blue staining demonstrates that dead cells can be stained, but living cells cannot **(E,G)**. **(H–J)** The root phenotype of GmCrRLK1L20-RNAi, EV-Control, and GmCrRLK1L20-OE under normal, drought, and salt stresses conditions, respectively. **(K)** Catalase (CAT) content of transgenic soybean hairy root composite plants and EV-control plants under salt and drought conditions. **(L)** Chlorophyll content of transgenic soybean hairy root composite plants and EV-control plants under salt and drought conditions. **(M)** Peroxidase (POD) content of transgenic soybean hairy root composite plants and EV-control plants under salt and drought stresses conditions. **(N)** Superoxide dismutase (SOD) content of transgenic soybean hairy root composite plants and EV-control plants under salt and drought conditions. **(O)** Proline (Pro) content of transgenic soybean hairy root composite plants and EV-control plants under salt and drought conditions. **(P)** Relative electrical conductivity of transgenic soybean hairy root composite plants and EV-control plants under salt and drought conditions. **(Q)** Malondialdehyde (MDA) content of transgenic soybean hairy root composite plants and EV-control plants under salt and drought conditions. **(R)** Fresh weight of roots of transgenic soybean hairy root composite plants and EV-control plants under salt and drought conditions. Vertical bars demonstrate ± SE of three biological replicates. ANOVA test indicated that there were significant differences (*p < 0.05, **p < 0.01).

Of these five genes, *GmCrRLK1L20* was notably upregulated under drought and salt treatment and was therefore used for further research (Figure 7). To further analyze the relationship between *GmCrRLK1L20* and stresses response in soybean, we conducted transgenic soybean hairy root composite plants to identify the plant stresses resistance and discovered that the overexpression of *GmCrRLK1L20* improved resistance to drought and salt (Figures 9A–C). The fresh weight of the roots was measured, which demonstrated that plants with *GmCrRLK1L20*-overexpression had higher root fresh weights (*GmCrRLK1L20*-OE) and that the *GmCrRLK1L20-*RNAi plants had lower fresh weights than wild-type plants (empty vector) under drought and salt stresses conditions (Figures 9H–J,R). These stressors can affect the intracellular reactive oxygen species (ROS) pathway which can produce H_2_O_2_ and O_2_^–^. Thus, we stained soybean leaves with 0.5% trypan blue and nitroblue tetrazolium (NBT) to analyze the H_2_O_2_ and O_2_^–^ contents of *GmCrRLK1L20*-RNAi, wild-type and *GmCrRLK1L20*-OE plants under normal or stresses conditions. The results demonstrated that there was no difference in trypan blue and NBT staining on the plant leaves under normal growth conditions (Supplementary Figure 1). However, the brightness of the leaf color in *GmCrRLK1L20*-OE plants was significantly dimmer than in wild-type plants, and the leaf brightness of *GmCrRLK1L20*-RNAi plants was significantly higher than that of wild-type plants under drought and salt stresses conditions (Figures 9D–G). We also measured several physiological and biochemical indexes related to stresses response, including CAT, POD, SOD, Pro, MDA, Chlorophyll, and relative electrical conductivity (Figures 9K–Q). These results demonstrated that the physiological and biochemical indexes of *GmCrRLK1L20*-OE and *GmCrRLK1L20*-RNAi plants did not differ from wild-type plants under normal conditions (Figures 9K–Q). However, the *GmCrRLK1L20-*OE plants experienced delayed leaf wilt (Figures 9B,C) and had longer roots (Figures 9I,J), higher CAT, POD, SOD, Chlorophyll, and Pro contents (Figures 9K–O), lower relative electrical conductivity, and lower MDA contents compared to wild-type plants under drought and salt stresses conditions (Figures 9P,Q). *GmCrRLK1L20*-RNAi plants experienced significantly increased leaf wilt (Figures 9B,C), had shorter roots (Figures 9I,J), lower CAT, POD, SOD, Chlorophyll, and Pro contents (Figures 9K–O), higher relative electrical conductivity, and a higher MDA content (Figures 9P,Q) compared to EV plants under drought and salt stresses conditions.

### Analysis of Molecular Mechanism of *GmCrRLK1L20* in Soybean

**FIGURE 10:**
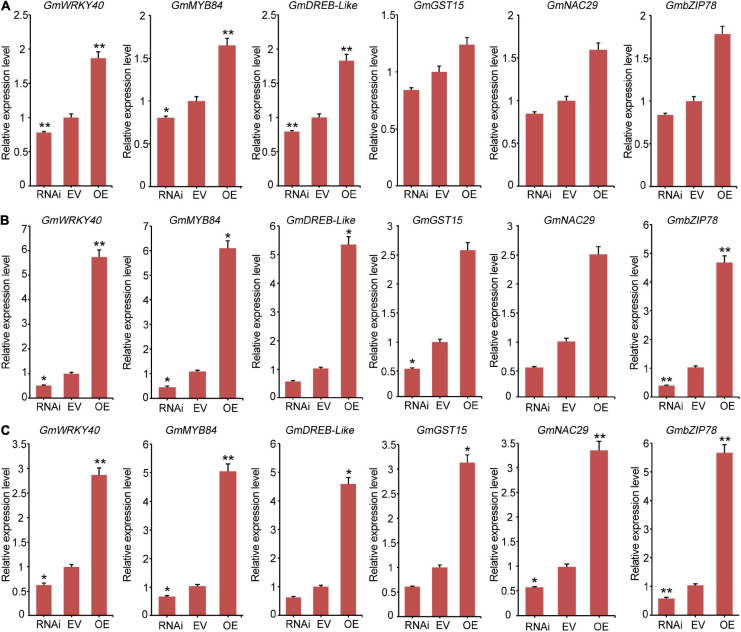
The expression levels of six stresses-responsive genes including *GmWRKY40*, *GmMYB84*, *GmGST15*, *GmDREB-like*, *GmbZIP78*, and *GmNAC29*. **(A)** The expression levels of six stresses-responsive genes were measured using qRT-PCR in transgenic *GmCrRLK1L20* soybean hairy root plants under normal conditions. **(B)** The expression levels of six stresses-responsive genes were measured using qRT-PCR in transgenic *GmCrRLK1L20* soybean hairy root plants under drought stresses conditions (200 mM mannitol). **(C)** The expression levels of six stresses-responsive genes were measured using qRT-PCR in transgenic *GmCrRLK1L20* soybean hairy root plants under salt stress conditions (200 mM NaCl). RNAi, EV, and OE represent *GmCrRLK1L20-*RNAi, EV-Control (the empty plasmid of pCAMBIA3301), and *GmCrRLK1L20-*OE, respectively. Vertical bars indicate ± SE of three biological replicates. ANOVA test indicated that there were significant differences (*p < 0.05, **p < 0.01).

To further analyze the molecular mechanisms of *GmCrRLK1L20* when subjected to abiotic stresses, we used qRT-PCR to assess the differential expressions of six stresses-responsive genes, *GmWRKY40*, *GmMYB84*, *GmGST15*, *GmDREB-like*, *GmbZIP78*, and *GmNAC29* in the *GmCrRLK1L20-*RNAi, EV-Control, and *GmCrRLK1L20-*OE plants. Several studies have found that these six stresses-responsive genes were either directly or indirectly involved in abiotic stresses (Marè et al., 2004; Xiang et al., 2008; Shahnejat-Bushehri et al., 2016; Sarkar et al., 2019). Meanwhile, the six stresses-responsive genes were found to be significantly upregulated in our *de novo* transcriptomic soybean sequences, and the value of log_2_ fold change (GH_treat/CK1_treat) and (NaCl_treat/CK2_treat) of the six stresses-responsive genes varied from 3.6 to 9.5 (Supplementary Table 2). qRT-PCR assays of six stresses response-related genes were conducted after the transgenic soybean hairy root lines and EV-control plants were treated with 200 mM NaCl and 200 mM mannitol, using corresponding untreated lines. A two-fold change in expression was considered an induction of expression. These results demonstrating that expression levels of the six stresses-response genes in *GmCrRLK1L20-OE* plants were significantly upregulated compared to the EV-control plants under drought and salt conditions (Figure 10) and expression levels of these six stresses-response genes in the *GmCrRLK1L20-RNAi* were significantly down-regulated compared to the EV-control plants under drought and salt conditions (Figure 10).

## Discussion

*Catharanthus roseus* RLK1-likes are a special subfamily of RLKs that are found only in plants. Previous research has demonstrated that CrRLK1L has one or two more carbohydrate-binding malectin-like domains than that of the subfamily of RLKs in ECLB (Boisson-Dernier et al., 2011; Lindner et al., 2012). We believe that CrRLK1L could have evolved special new adaptations for their environment. CrRLK1L also plays an important role in growth, development, and stresses response in certain plants (Lindner et al., 2012). However, the biological and genetic roles of CrRLK1L in soybean are still unknown. By analyzing the structural characteristics of *CrRLK1L* genes in soybean, we found that the majority of soybean *CrRLK1L* genes lacked introns and that individual members have a short intron in the 5′UTR (5′untranslational region) or 3′UTR, but that no introns exist in both the 5′UTR and 3′ UTR (Figure 3). This indicates that these genes rarely exist in the form of alternative splicing (Li et al., 2015). The fact that most soybean *CrRLK1L* genes lack introns could be due to selection pressures during the evolutionary process. However, several soybean CrRLK1L genes have evolved a distinct exon-intron structure with 1–10 introns, and we discovered that the soybean *CrRLK1L* genes that contained similar gene structure and motif elements were divided into the same subfamily (Figure 3). We conducted an additional analysis of the phylogenetic tree of *CrRLK1L* genes in *Arabidopsis*, rice, and soybean according to their conserved amino acid sequence (Figure 1). The 71 *CrRLK1L* genes were classified into three clusters, while a majority of soybean *CrRLK1L* genes are distributed in cluster II. Five CrRLK1L family members from the *de novo* transcriptome soybean sequencing were classified into group II, with the exception of *GmCrRLK1L24.*

Analysis of the phylogenetic tree demonstrated that CrRLK1Ls shared high homology with *Arabidopsis* FER (Figure 1). The available evidence suggests that FER plays a vital role in hormone response (Hematy et al., 2007; Hematy and Hofte, 2008; Guo et al., 2009), cell growth, and abiotic stresses in plants (Huck et al., 2003). For example, FER participates in the identification between pollen tubes and its subsidiary cell (Boisson-Dernier et al., 2009), the elongation of pollen tubes and nutrient tissue cells, and the regulation of stomatal aperture (Rotman et al., 2003; Guo et al., 2009; Kessler et al., 2010). FER can regulate the production of ROS and calcium, which serve as vital second messengers in abiotic and biotic stresses responses (Luan et al., 2009; Gilroy et al., 2016). Based on the above analysis, we speculate that CrRLK1L family members could participate in hormone responses, and abiotic stresses. Further analysis of the *cis*-acting elements for the promoter of soybean *CrRLK1L* genes revealed that the 38 soybean *CrRLK1L* family members were highly correlated with stresses responses, such as ARE, ABRE, MYB, MYC, LTR, WUN-motif, MBS, DRE, TC-rich, and W-Box (Walker and Zhang, 1990; Walker, 1994; Chinnusamy et al., 2004; Shiu et al., 2004). This indicates that soybean *CrRLK1L* family members could play an important role in regulating stresses responses.

In our study, the method of the rapid drought that soybeans were placed on filter paper and the salt stress treatment that soybeans were immersed into 200 mM NaCl solution were used to preliminary screen the vital genes that rapidly respond to drought and salt stress, and this method of stress treatment can only be used in the laboratory and cannot be applied to agricultural environment or nature environment (Chen et al., 2015; Du et al., 2018). In addition, some studies have indicated that the methods for assaying plant resistance, on artificial media, filter paper and in hydroponic systems conditions, does not accurately mimic what is observed when plants are grown in soil (Rich and Watt, 2013; Robbins and Dinneny, 2015; Nelson and Oliver, 2017). Therefore, the method of the rapid drought and salt were used to initially screen the vital gene responding to drought and salt stress by qRT-PCR in our study. qRT-PCR analysis also found that drought and salt stresses could affect the expression levels of five *CrRLK1L* genes from *de novo* transcriptome sequencing of soybean. *GmCrRLK1L20* had the highest transcription levels of the five genes under stresses conditions (Figure 7), which was consistent with the hierarchical clustering of expression profiles from drought and NaCl- RNA-seq (Figure 6). *GmCrRLK1L20* was therefore selected for further analysis of its gene function under drought and salt stresses. Its molecular features indicated that the GmCrRLK1L20 protein is localized in the cell membrane. To further verify the interactions between the *GmCrRLK1L20* gene and plant resistance, we generated transgenic soybean hairy root composite plants (RNAi, EV, and OE) via the *Agrobacterium*-mediated method and conducted nature drought and salt treatment for this composite plants, and we discovered that the *GmCrRLK1L20*-overexpression plants enhanced drought and salt tolerance compared with wide-type plants (Figures 9B,C). This suggests that the overexpressed plants had longer roots than the RNAi and EV plants (Figures 9H–J), had higher CAT, POD, SOD, and proline contents, and had lower relative electrical conductivity and a lower MDA content than RNAi and EV plants under drought and salt stresses (Figures 9K–Q). This demonstrates that *GmCrRLK1L20* provides effective resistance against some abiotic stressors.

In order to analyze the molecular mechanism of the *GmCrRLK1L20* gene, we selected the stresses-response genes *GmWRKY40*, *GmMYB84*, *GmDREB-like*, *GmGST15*, *GmNAC29*, and *GmbZIP78* for additional study (Figure 10). GmWRKY40 is a WRKY transcription factor and can enhance the expression of downstream stresses-related target genes by specifically binding to the W-box. For example, researchers have demonstrated that WRKY transcription factor genes (*OsWRKY11*) can directly bind to the promoter of the stresses-responsive gene *RAB21*, and can enhance stresses tolerance in transgenic rice seedlings (Marè et al., 2004). GmMYB84, an R2R3-MYB transcription factor, can improve the expression levels of *GmRBOHB-1&2* genes by binding to the MBS cis-elements in their promoter, enhancing stresses tolerance in soybean (Wang et al., 2017). GmDREB-like, a DREB-type transcription factor, can specifically recognize and bind to the DRE/CRT *cis*-acting element to regulate the expression of downstream stresses-responsive genes such as *RD29A*, which improves plant stresses resistance (Sarkar et al., 2019). GmNAC29, a NAC-type transcription factor, can recognize the CATGT and CACG elements of various downstream stresses-responsive gene promoters involved in plant stresses resistance (Tran et al., 2004). For example, the *Arabidopsis* NAC transcription factor *JUB1* promotes stresses response by directly repressing the expression of *GA3ox1* and *DWF4* genes (Shahnejat-Bushehri et al., 2016). GmbZIP78, a bZIP*-*type transcription factor, can recognize ABRE responsive elements in stresses-responsive gene promoter regions to regulate the expression of ABA-related pathway genes. This improves sensitivity to ABA, which could enhance plant tolerance to drought and salt stresses (Xiang et al., 2008). GmGST15, a glutathione S-transferase protein, encodes a ROS-scavenging enzyme and regulates the homeostasis of ROS in cells. The ROS-scavenging enzyme was related to maintaining cell redox homeostasis and protecting cells from oxidative stresses under stressful conditions (Jha et al., 2011; Qi et al., 2018). Our results demonstrated that these stresses response genes were significantly upregulated in transgenic *GmCrRLK1L20* soybean hairy root composite plants. This indicates that *GmCrRLK1L20* improved soybean resistance to abiotic stresses by regulating the expression of several stresses response genes and playing a critical role in plant resistance to drought and salt stresses. These results indicate that the *CrRLK1L* gene is involved in plant stresses resistance and provides a theoretical basis for the additional study of plant production.

## Conclusion

In this study, we identified 38 *CrRLK1L* genes in the soybean genome sequence. We conducted a comprehensive and systematic analysis of structural features and expression profiles and performed phylogenetic analyses. Finally, we identified a *GmCrRLK1L20* gene that was significantly upregulated under drought and salt stresses conditions. These results provide insight into understanding the evolutionary mechanism of *CrRLK1L* genes and how they relate to stresses response in plants.

## Data Availability Statement

The datasets presented in this study can be found in online repositories. The names of the repository/repositories and accession number(s) can be found below: NCBI SRA [accession: PRJNA694374].

## Author Contributions

Z-SX coordinated the project, conceived and designed experiments, and edited the manuscript. Z-QW performed experiments and wrote the first draft of the manuscript. T-FY revised the manuscript. JC, Y-BZ, and MC contributed to data analysis and managed reagents. G-ZS, W-LW, and Y-ZM contributed with valuable discussions. All authors reviewed and approved the final manuscript.

## Conflict of Interest

The authors declare that the research was conducted in the absence of any commercial or financial relationships that could be construed as a potential conflict of interest.

## Footnotes

1 http://arabidopsis.org

2 http://www.phytozome.net

3 http://smart.embl-heidelberg.de/

4 http://web.expasy.org/protparam/

5 http://gsds.cbi.pku.edu.cn

6 http://meme.nbcr.net/meme/intro.html

7 http://bioinformatics.psb.ugent.be/webtools/plantcare/html/

## Supplementary Material

Supplementary Figure 1 The trypan blue and NBT staining of plant leaves under normal growth conditions.

Supplementary Table 1 Primers and sequences used in this study.

Supplementary Table 2 The value of log_2_ fold change of the six stresses-responsive genes from the *de novo* transcriptomic sequences of soybean.

Supplementary Table 3 The expression data of the five soybean *GmCrRLK1L* genes under drought and salt treatment.

## Abbreviations

RLKs: receptor-like protein kinases
PKC: protein kinase catalytic domain
ECLB: extracellular ligand-binding domain
TM: transmembrane domain
ABA: abscisic acid
ABRE: ABA-responsive elements
FW: fresh weight
GSDS: gene structure display server
LTR: low-temperature responsive
MBS: MYB binding site
NCBI: National Center for Biotechnology Information
qRT-PCR: real-time quantitative polymerase chain reaction.
