# Supplementary figures and images for: Genome-Wide Analysis of the Catharanthus roseus RLK1-Like in Soybean and GmCrRLK1L20 Responds to Drought and Salt Stresses
Source: Front Plant Sci. 2021 Mar 18;12:614909. doi: 10.3389/fpls.2021.614909 (PMC8012678; doi:10.3389/fpls.2021.614909)

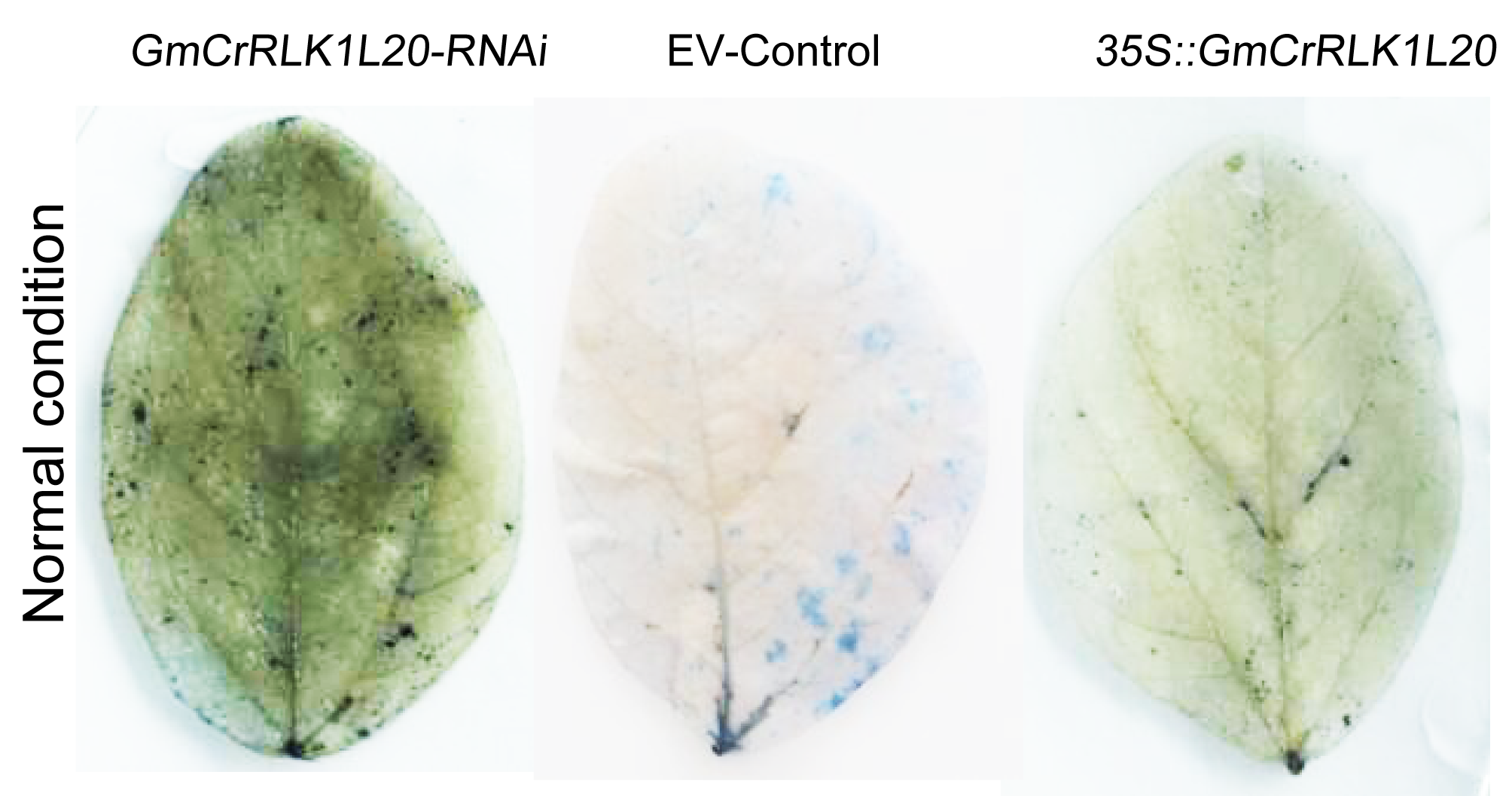

Supplement: Supplementary Figure 1 — The trypan blue and NBT staining of plant leaves under normal growth conditions. [file Image_1.tif]

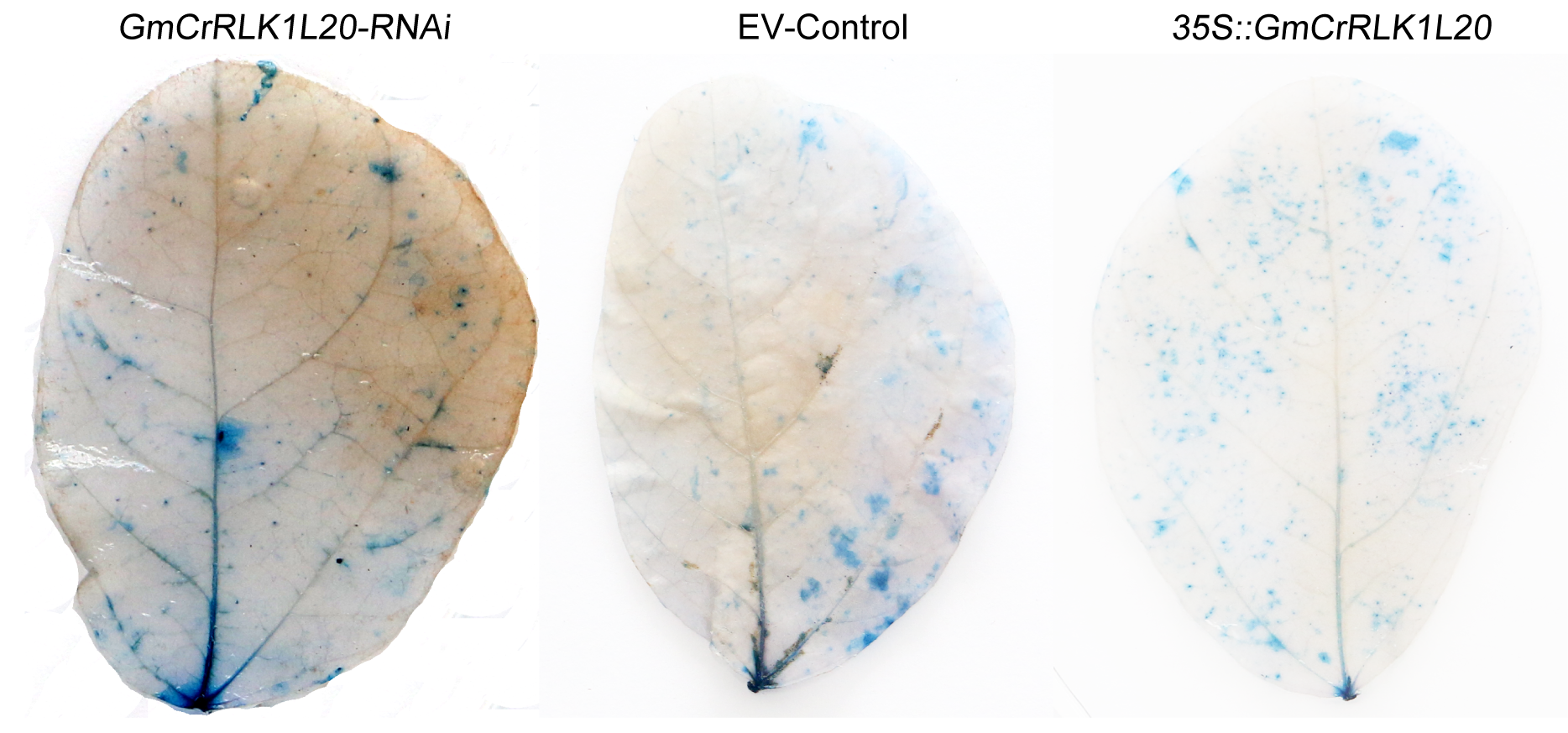

Supplement: Supplementary file 6 [file Image_2.TIF]
