# Supplementary material for: Genome-Wide Analysis of the Catharanthus roseus RLK1-Like in Soybean and GmCrRLK1L20 Responds to Drought and Salt Stresses
Source: Front Plant Sci. 2021 Mar 18;12:614909. doi: 10.3389/fpls.2021.614909 (PMC8012678; doi:10.3389/fpls.2021.614909)
Supplement: Supplementary Table 1 — Primers and sequences used in this study. [file Table_1.docx]

**The primers used in this study are as follows:**

GmCrRLK1L20 (16318GFP)-F: TATCTCTAGAGGATCC ATGAGGTTCAACAGTATCACC

GmCrRLK1L20 (16318GFP)-R: TGCTCACCATGGATCC GTAGTGAGTTGAGTATGACT

GmCrRLK1L20 (3301)-F: GGACTCTTGACCATG ATGAGGTTCAACAGTATCACC

GmCrRLK1L20 (3301)-R: ATTCGAGCTGGTCACC GTAGTGAGTTGAGTATGACT

GmCrRLK1L19-F: GTGTGACTACGACTTCGACTAT

GmCrRLK1L19-R: TTAATCAATTCACTCAACGCGG

GmCrRLK1L20-F: CAATGCATCCTTCACTGTTCAA

GmCrRLK1L20-R: TTCGTGTTTTCCGCTTTTAGAG

GmCrRLK1L22-F: CAGGAAGAAAGGTCCTTGTTTG

GmCrRLK1L22-R: TATCCTGGAGTAGTTGCTAGGA

GmCrRLK1L24-F: GAGCAACTACGAGTACTTCAGT

GmCrRLK1L24-R: CAGCACGCAAATTGTACTTTTC

GmCrRLK1L31-F: CTATTTCTCAGTCAAAGCTGGC

GmCrRLK1L31-R: CCATTGATGAAAGCGTACGAAT

**Gene sequence:**

>Glyma.13G053800.1 CDS
ATGACGTTCCTCAGTATCACCTCCTTAACACTATTCTTCCTTCTTCTCTTTCTCTCCATAGAGCTCCAAGCGTACACCCCAGAAGACAACTTCACCATCAGTTGTGGCACCACCGGGATAGTCTTCGACGGCCAAAGGACATGGACGGGGGACGCAGACACAAAGTACTTATCTGGTGGTCAAGGCAGCACCGTTTTAACCCAAGCAGCAACACAAGATCCTTCTGTCAATCAAGTTCCCTACACCACAGCACGGTTATCCCCTTCTCAGTTCAATTACTCCTTCCCCGTTTCTGCGGGCCCCAAATTCGTTCGCCTCTTCTTCTACCCGGCTGATTACCCTTCCTTTCCCCGCACTGATGCCTCTTTCTCCGTTCAATCTAACGGATTCACCTTCTTAAAAGGTTTCAACGCCTCTCTAAACGCTGACGCGGAAGCCACCAAAACCATCTTCAGGGAATATGTGGTCAACGTCAACGACGGTGAGACTCTCATCCTCAGCTTCACTCCCTCGCAACCAAACTCCTACGCTTTCATCAATGGAATCGAGGTGCTTTCCATGCCAAGTGATTTGTATTACACGTCAGCAACTGACTCAACAGGATTCAAGTTCTTGGGAAGTACCACGCTGTACAGCGTAGAAACCAGATTTGCGCTGCAGGCGGAGTATAGAATCAAAATGGGAGGGCAAGAAATATCACCACTAAACGACACCGGTTTGTTCAGGAAATGGGCCGATGACGAAGAAGATTATTTAATCAAACAAAATCCACAGAATAATGATCTTTCAAGCAACACGGATGGTAAGATGAACATAACCGTGAATCCTGATTACGTGGCACCCAAGGAACTCTACAGAACAGCGCGTAACATGGGCACAAACGCCACTCTGAACAAAATCAGCAACCTGACTTGGGAGTTCCCGGTTGATTCTGGCTTCACTTACGTTCTCAGGCTTCACTTTTGCGAGCTTGACCCCAATATTAATAAAGATGGTGACAGGGTGTTCTTCATTTACATAGCGAGCCAGTTGGCTGAGAACCACGCTGATGTTATGCAATGGAGCCACAATCAGAAAGGTCTCGCTGTGCAGAGAAACTATGCCGTTTTAATTCCGAAGGACAATACTCAGAAAAAGGTTAATCTCTCGCTTCAGATGCATCCTTATGCAACTAATGATGAAACGACATACAGCGACGCGTTCTTGAACGGTCTCGAGATCTTCAAAATCAGTGAGGCTGGGTCAAACAACCTTGCCGGACCTAACCCGGACCCGGTTCAGACTCCACACAACAACATACCCGCCCCAAAGGGAAACCGCAGCAGCAAAAGCGGAACGTCGATAATCGGCATCGTGGCAGGTGTGGTATCCGGCGTCGTTTTGATCTCACTCATCATCCTTTTCCTCATCGTCTTCTTCCGACGCAAGACAATCACTACGCCCAAGGACTACAACAAGTCCAAGTCCTCCGCGACCTCCAAGTGGGGCCCACTCTCCTTCACAACGACCAAGTCAACCACCACCACGAAGTCCTCCCTCCCCTCCGAACTCTGTCGTAACTTTTCCCTCGCCGAGATCGAAGCCGCCACCAACAACTTCGACGATGTTCTCATCATTGGCGTCGGGGGATTCGGCCACGTGTACAAGGGCTACATCGACGGCGGTTTCACCCCAGTCGCCATCAAGCGCCTCAAACCGGATTCACAGCAGGGTGCAAATGAATTCACGAATGAGATCGAGATGCTCTCACAGCTACGCCACCTTCATCTCGTGTCCCTCATTGGGTACTGCAACGAGAACTACGAAATGATCCTCGTCTACGATTTCATGGCACGTGGCACCCTCCGGCAACATCTCTACAACTCTGATAACCCTCCTGTCTCGTGGAAGCAGCGCTTGCAAATTTGCATTGGTGCCGCACGTGGGCTGCATTATCTCCACACAGGCGGGAAGCACACAATCATCCACCGTGACGTGAAAACCACCAACATACTGTTGGATGACAAGTGGGTGGCCAAGATTTCCGACTTTGGGCTATCCAGAATTGGGCCCACGAGCATTGACAAATCTCATTGCTCTTTGAGATATTGTGCGCTCGGCCACCTCTAA

>Glyma.13G054200.1 CDS
ATGAGGTTCAACAGTATCACCTCCGTAACAACACTCTTCCTCTTTCTCAGCCTCTTTACACACCTCCATGCGTACACTCCCGTGGACAACTTTACCATAAGCTGCGGCACTACCGGAAAATCTTACGACGGGGAAAGGACATGGACGGGGGACACAGGTTCAACGTTCTTATCTCATCAAGACGGCACCGTTTCCGCTAATGCAACAACACAATCTCCTTCTACCAATCAAGTGCCCTACACCGCCGCACGCTTGTCCCGTTCCCGTTTCAGCTACTCCTTCCCAGTCTCCCCTGGCCCCAAATTCCTCCGCCTCTTCTTCTACCCTGCTGAGTACGCTTCCTTTCCTAGCTCCAATGCATCCTTCACCGATCAATCCAACCAATTCACCCTTCTCCACGTTTTCAATGCCTCTCTAAAAGCGGAAAACACTAAAACCATCTTCAGAGAATATGTCGTCAATGTGGACGGCGACAGTGAGAGGTTGAACCTCACCTTCACTCCGTCACAACCAAACTCCTACGCTTTCATCAACGGAATCGAGGTACTCTCCATGCCAAGCAATCTGTATTACACCTCAGCAAATGACAACGGTTTGAAGCTCGTGGGAACTGACACACTGTTCCCCATAAGAACCAACACTGCACTGGAGACAGAGTATAGAATCAAAGTGGGAGGGCAAGGAATCTCGCCACGAAACGACACTGGTTTGTTCAGGAACTGGGCTGGTCAAGATGAAGATTATTTAATTAAACAAAAAAATCCACAGAATAGTGCTATAACAGGAAACACCAATGGTAAGATGAACATAACGGTAAATCCAGATTATGTGGCACCCAAGGAACTGTACAGAACAGCTCGTGTCATGGGCACAAACACGTCTATGAACAAAAGCCTCAAGTTGACTTGGGAGTTCCCTGTTGACTCTGGCTTCCACTACATGATCAGGTTCCACTTTTGCCAACTTGACCCCAATATTACTAACATCGGTGACAGGGTGTTCTCACTTTACATAGGGAGCGAGTTTCTTGATGTTATGAGATGGAGCCAAAAACAGAAAGGTGTCGCTGTGTACAAAGACTACGCCATTTTAATCCCCAAGAGTGATACTCAGAAACAGGTTAATCTCTCGCTCCAAATGATGAATCCTTATGAAAGTGCCAAGGATAAAGAAAACAATGACCCGTTTCTGAATGGTCTGGAGATCTTCAAAATCAGCGAGTTTAACAACCTTGCTGGACCTAACCTGCAGAACAACAACATGCTCGTCCAAGAAGGAAAGAACAGCAGCAGAACACTTAAAATTGTTGTAGCAGGGGTGGTATCTGGCGTTGTTGTTTTCTTCATCTTTTTGTGGGGGAGTTGCAAGTTCGGTCCTCTCTTGCCCTCGCAGGATGACGACATGCTGAATTACAGCCAGAGGTGGCCGTTCAATCTCCTCTGCCAGCGCTTTTCCCTCATGGATATCAAAGCCGCCACCAACAACTTCAATAACGAATCCCTCGTCGGTGTTGGAGGATTCGGCCACGTGTACATGGGCTACATTGACGGCATTTCCATCCCGGTGGCTATCAAGCGTCTCAAGCCGGGTTCGAAGCAGGGGTCCGAAGAGTTCCTGACCGAGATCAAGATGCTCTCGCAGATCCGCCACCGCCATCTCGTTCCTCTCATCGGCTACTGCAACAACAACAAGGAGATGATCTTGGTCTATGATTTCATGACACGTGGCAATCTCCGTGACCATCTCTACAACACCGACAAATCCCCTTTATCGTGGAAGCAACGTTTGCAGATTTGTATTGGCGCCGCCCATGGGTTGTATTATCTTCACAAATGTGCCGGGAAATACATGATCATCCATGGTGATGTGAAAACCACCAACATCTTGTTGGATGATGACTGGGTGGCCAAGGTTTCGGACTTTGGGCTTTCCAGATTTGGGCCCACGGACTCATCCCATGCCTATGGCAGCACCACCGCTGTAAGAGGCAGCTTTGGGTATATAGACCCAGAGTATTATAAGCGGCATCATTTGACGGACAAGTCTGACGTGTACGCTTTCGGGGTAGTGTTGTTTGAGGTACTGTGCGCTCGTCCGCCTCTTATACGCAATGAAGATCCCAAACAGGAGTCGCTTGCTAAGTGGGTTAGGTACTGTTACCAAAGTGGGACCATGGACCAGATTGTGGACCCCACGTTGAAGGGGAGGATCGCGCCTGAATGCTTCCGGAGGTTTTGCCATATTGGGGTGAGTTGTTTGTCAGAGGTTGGGACGCAGAGGCCGTCGATGAAAGATGTTGTTTTCATGTTGGAGTCTACTCTGCAGGTGCAAGAGAGCGCGGAGAATGTAAAAAGAGGAAATTAG

>Glyma.13G054400.1 CDS
ATGAGGCTCCTTAGCATCATCACCACCTCCTTAACACTATTCTTCCTTCTTCTCTTTCTCTCCATAGAGCTCCAAGCGTACACCCCAGAAGACAACTTCACCATCAGTTGTGGCACCACCGGGATAGTCTTCGACGGCCAAAGGACATGGACGGGGGACGCAGACACAAAGTACTTATCTGGTGGTCAAGGCAGCACCGTTTTAACCCAAGCAGCAACACAAGATCCTTCTGTCAATCAAGTTCCCTACACCACAGCACGGTTATCCCCTTCTCAGTTCAATTACTCCTTCCCCGTTTCTGCGGGCCCCAAATTCGTTCGCCTCTTCTTCTACCCGGCTGATTACCCTTCCTTTCCCCGCACTGATGCCTCTTTCTCCGTTCAATCTAACGGATTCACCTTCTTAAAAGGTTTCAACGCCTCTCTAAACGCTGACGCGGAAGCCACCAAAACCATCTTCAGGGAATATGTGGTCAACGTCAACGACGGTGAGACTCTCATCCTCAGCTTCACTCCCTCGCAACCAAACTCCTACGCTTTCATCAATGGAATCGAGGTGCTTTCCATGCCAAGTGATTTGTATTACACGTCAGCAACTGACTCAACAGGATTCAAGTTCTTGGGAAGTACCACGCTGTACAGCGTAGAAACCAGATTTGCGCTGCAGGCGGAGTATAGAATCAAAATGGGAGGGCAAGAAATATCACCACTAAACGACACCGGTTTGTTCAGGAAATGGGCCGGTGACGAAGAAGATTATTTAATCAAACAAAATCCACAGAATAATGATCTTTCAAGCAACACGGATGGTAAGATGAACATAACCGTGAATCCTGATTACGTGGCACCCAAGGAACTCTACAGAACAGCGCGTAACATGGGCACAAACGCCACTCTGAACAAAATCAGCAACCTGACTTGGGAGTTCCCGGTTGATTCTGGCTTCACTTACGTTCTCAGGCTCCACTTTTGCGAGCTTGACCCCAATATTAATAAAGATGGTGACAGGGTGTTCTTCATTTACATAGCGAGCCAGTTGGCTGAGAACCACGCTGATGTTATGCAATGGAGCCACAATCAGAAAGGTCTCGCTGTGCAGAGAAACTATGCCGTTTTAATTCCGAAGGACAATACTCAGAAAAAGGTTAATCTCTCGCTTCGGATGGATCCTTATGCAACTAATGATAAAACGACATACAGCGACGCGTTCTTGAACGGTCTCGAGATCTTCAAAATCAGTGAGGCTGGGTCAAACAACCTTGCCGGACCTAACCCGGACCCGGTTCAGACTCCACACAACAACATACCCGCCCCAAAGGGAAACCGCAGCAGCAAAAGCGGAACGTCGATAATCGGCATCGTGGCAGGTGTGGTATCCGGCGTCGTTTTGATCTCACTCATCATCCTTTTCCTCATCGTCTTCTTCCGACGCAAGACAATCACTACGCCCAAGGACTACAACAAGTCCAAGTCCTCCGCGACCTCCAAGTGGGGCCCACTCTCCTTCACAACGACCAAGTCAACCACCACCACGAAGTCCTCCCTCCCCTCCGATCTATGCCGCCACTTCTCCCTCCCGGAGATCAAGTCCGCCACCAACAACTTCGACGACGTCTTCATCGTCGGCGTCGGCGGATTCGGCCACGTGTACAAAGGCTACATCGACAACGGCTCCACCCCCGTCGCCATCAAGCGCCTCAAGCCGGGTTCACAGCAAGGCGCGCACGAGTTCATGAACGAGATCGAGATGCTATCGCAGCTCCGCCACCTCCACCTCGTATCTCTCATAGGTTATTGCAACGAGAACAACGAGATGATCCTCGTCTACGACTTCATGGCGCGTGGAACGCTACGCGATCATCTATACAACACCGACAACCCCCCTTTGACGTGGAAGCAGCGCTTGCAGATCTGCATCGGCGCCGCGCGTGGACTGCATTACCTCCACACCGGCGCGAAGCACACGATCATCCACCGCGACGTGAAAACTACCAACATTTTGTTGGATGATAAGTGGGTGGCCAAGGTTTCGGACTTCGGGCTTTCGAGAATCGGGCCCACGGGCAACGCCAAGGCCCACGTCAGCACCGTTGTGAAAGGCAGCATTGGGTATTTGGACCCGGAGTATTATAAACGACAGCGTTTAACTGAGAAATCTGACGTGTATTCCTTTGGAGTGGTGCTGTTTGAGTTACTCTGCGCTCGTCCGCCTCTGATCAGAACTGCGGAGAAGAAACAGGTGTCGCTTGCTGATTGGGCGAGGCACTGCTGCCAAAATGGGACCATAGGCCAGATTGTGGACCCCACTTTAAAGGGGAGGATGGCGCCAGAGTGTTTGAGGAAATTCTGCGAGGTTGCGGTGAGTTGTTTGTTGGACGACGGAACGCTGAGGCCGTCGATGAACGACGTCGTTTGGATGCTGGAGTTTGCGTTGCAGTTGCAGGAGAGTGCTGAGCAGCGTGAAAATACTAATATTGTTGATAATGAAATTAATGAGAGAAGAGAGGAGGAGGCTAGTGATGATTTGTTTAGTACTGGAACCAGTGTGGGCCAGGTTTCGGATTTTAACAAGAGTAGCGGTGTGGTGAGTGTGAGTACTGATAGCGAAGAGCTTAGTAGTAGCTACAAAGAGAGTGATAAGTTGATGTCTGGGACTGTTTTCTCTGAGATTGTGGATCCAAAGCCACGTTGA

>Glyma.15G042900.1 CDS
ATGACTGATTGGAGAAATATTGGTTTCTTTATATGTGTTTTATCAATCTTTCCGCTTGTATGCTTCTGTGCCACCTTTGTTCCAGTAGATAATTATCTTATAGACTGTGGAGCAACTACGAGTACTTCAGTAGGTACACGCAATTTCATAGCAGATAACAAGGATCTCCTTTCCACACAAAAAGATATTGTTGCCACTACCTCCTCAAAATCAGCTACTTCTTCCTCTGATGATTCATCGCTCTATCAAACTGCAAGAGTCTTCACTGCTTCCTCGAAGTACACTTTTAAAATTAATCAAAAGGGGAGGCACTGGATCCGTCTCTATTTCCTTCCATTCGCTTATGAAAAGTACAATTTGCGTGCTGCAGATTTCACTGTTTCCACCCAAAACCATGTCCTTTTCAGAAGCCTCAACATGCAGAAAGATCCTGTGATGAAGGAGTACTCAGTGAATGTGACCTCCGACTCCCTTGTTCTTACCTTTGCCCCTTCAGGAAGTTCCATTGCTTTTGTGAATGCCATTGAAGTTGTTTCTGTCCCTGATGACCTAATTGTTGATGATGGTTTTGCCTTAGATCCATCAGTAACCTCTTCTGGGTTGGTGACACAAGCACTTGAGACAGTTTGGAGGGTTAACATGGGTGGTCCTACTGTGACCCCTATAAATGACACCCTTCAAAGAACTTGGGTTCCTGATCAAAGTTTCCTTTTGCAATCCAACCTTGCCTCATTTTCTTCCAATATTAAGGGTGTTAAGTATGAGAACCATGGGCAAGCAACAGAAAACACTGCTCCGCCTACCGTTTATGGGACTCTCACACAGATGAACTCGACCTATGATCCCCGTAATATTTTCAATGTAACATGGCAGTTTGATGTAAGTCCCGGATTTCAGTACCTTGTTCGACTTCACTTCTGTGATGTAGTCAGTAAAGCTCTCAATGAACTCTACTTCAATGCTTATGTTGACTCCAAACTGGCTGCTTCAAGTGCTGATCCCAGTACTACTAGCAATAATGCTTTGGGTGTTCCATACTATAGGGATCTGGTTACAGCCGTGGCTGTCAGCAAAACACTTCGTGTAAGTATTGGCCCTTCTGAGGTAAATAAGGAGTACCCTAATGCCATTTTGAATGGGTTGGAGATCATGAAAATGAACAATTCTATGGGCAGTCTTATCCCAGGAGCTGTGGCTATTACTTCTGGCTCAAGTTCTAAAAAAACTGGCATGATTGTGGGTGTGAGTGTTGGGGTAGTTGGTGCAGTTGTCTTGGCTGGAGTTTTCTTTGTACTGTGCAGGAAAAGAAGAAGGTTGGCACAAAGGCAGTCAAAGACATGGGTTCCTTTATCCATCAATGATGGAACTACTTTTCATACCATGGGAAGTAAATATTCTAACGGCACAACACTGAGTGCTGCTTCAAACTTTGAGTACCGAGTCCCTTTTGTGGCGGTTCAGGAGGCTACAAATAATTTTGATGAGAGTTGGGTTATTGGCATTGGTGGTTTTGGTAAAGTGTACAAGGGAGAGTTAAGTGATGGCACAAAAGTGGCAGTCAAGAGGGGGAATCCACGATCACAGCAGGGGCTTGCGGAGTTCCAAACTGAAATTGAAATGCTTTCTCAATTCCGTCATCGCCATCTAGTGTCTTTGATTGGTTATTGTGATGAAAGGAATGAAATGATCTTGATATATGAATATATGGAGAAGGGTACTCTCAAGGGTCATTTATATGGTTCAGGTCTGCCAAGCTTAAGCTGGAAGGAGAGGCTTGAGATATGCATTGGAGCAGCTAGAGGGCTTCATTACCTTCACACTGGCTATGCTAAAGCAGTTATTCACCGCGATGTGAAGTCTGCAAATATCCTACTTGATGAGAACCTGATGGCTAAAGTTGCTGATTTTGGATTATCAAAGACGGGGCCTGAAATTGACCAGACACATGTTAGCACAGCTGTTAAAGGTAGTTTTGGGTACCTTGATCCGGAGTATTTTAGGAGGCAACAACTAACAGAAAAGTCAGATGTATATTCATTTGGGGTAGTTCTGTTTGAAGTTCTTTGTGCAAGGCCTGTCATAGATCCAACACTTCCTAGAGAAATGGTAAACTTGGCAGAATGGGCGATGAAATGGCAGAAGAAAGGACAGTTGGAGCAAATCATAGATCAAACACTTGCAGGCAAAATAAGACCAGATTCTCTAAGGAATGATCTTCATATATGA

>Glyma.18G270600.1 CDS
ATGGTACCCCCAGTTATTCACCAAATCTGCTTTGGTCATTCCAATGCCGCGGTCAATAATGGAAAGAGTCTTGTTGACTTTGTCGAGCACAAGACGGATGAAGAACTCTGGTTGAGCATCGAGCTTGCTCTTGTCAGAATGATCCTCCACATTTTTTACTGCAACAACGGTGTAATCTCCTTGAGGCGGTGGCTTCAGAGGTGGTGGTGGGCGTCGACACCGGTGAGGAACAGAACAAAGCTGAGTCATAGTGGAGAGAACCCAAACCCAACGCGGATGAATCTGGTGCGTGACGGCGACGTCCCCGAGCAGGATGATGTCGTTGTCGTGGAGGATCTTGCGGTAACGACGGTGGTTGGTGCGGAGCAGCAGAGAGTGACTTTTCAAGTCGTACAAGAGGGTGTAATTCTAGAAAGGTTCTCTCAGTGTAGTGGGAATCTTATACAGCGATCTGGGTTGTTTTATCCTGGGGACTTCATGGTTGATAGTCTGCTTGCACAATTTTTGTGCAATGCCACGAAACGTTTTAAAGAAAGTGACATTGTCTGCGACTCAACCAAGATCGAGTTTGAAGAGCATGGAGGCGTGGAACGCGTCAAGACGAAGTTGAGATCCATGGAGAGCGGAATCCATGGAAGCTTGGGTTGCGGCGAGGTTGGCCATGGCGGCCTCGAGGCGGTCCAGGCTGGCGAAAGGTTCG
ACATCTGCCATGGAAGAGCGACAGAGAAGGGGGCAGCCTTATCGATGGACACCACTGACACACGAATTGCTTTAGGATCCATCCTCATCCTCCTTTTGATGTGTCTCTCTAACCTTTCAATAGCAGAGGTTATTTATTCTCCAGATGAGCTCTTGAGCATCAACTGTGGCTCTTCCTCCAACTTTTCCAGTCGCGATGGTCGCAACTGGACTGTAGACATCAATTTCCTCATAGCTGAAAGCCGTGTCAACTCGGTTGCTGCCCCTGCACTTACTCCAACTACCCAAGAGGGTCCTTACACCTATGCTCGTCTATCTCGCTCCCAATTCACTTACTCGTTCCCCGTCACTGCAGGCCCAAAGTTTCTTCGCCTCTTCTTCCACTCAACTTCATACCATAACTTTGATCGCCCCAATGCCTATTTCTCAGTCAAAGCTGGCTCATACACCCTCCTTCGAGATTTCAATGCTTCCCTCAATGCTGATGCTGATGACGGCCCTGGCCAGACCGACATCTTGTTCAGAGAGTATTGCATCAACCTTGAAGATGGTCAGAAGCAGCTCAACATAACCTTCATTCCAAGCAAAACAGCTCAACATCCATATTCGTACGCTTTCATCAATGGAATCGAGATTGTTTCAATGCCCCCTTATCTCTACTACACCAATCCTGACGTTGACATCTCTGGAGAGCCGCAAATTGTTGGAGGAGGAACATCTACTATTGAAAATAACTCAGCTCTCGAGACAATGTACCGGTTGAATGCTGGAGGACGAACAATCCCTTCATCAGAGGACACAGGTATGCTCAGGACCTGGGATGCCGACGACAAATACCTAACTATTAAACCTACATCATTATCTGTTGATTATGGGAAGAGTACTAAGCTCAGCTTTACTGCTATGGTTCCTAACTACACGGCACCAGACGAAGTGTACCGAACCGTAAGGGGTATGGGAACGAATTTTTCCGTCAACATGGGGTTCAACCTCACATGGAAGCTTCCCGTTGATTCTCGCTTCACCTACTTGTTTAGGTTACACTTTTGCCAGCTTGATCCACAAGTTACGGATGCTGGCGACCTAGAATTCTACATCTACATAGAGGATCAGTTGGTTAACGATAGGGCAGACGTTCTCTTTTGGACCGATAACCAGAAGGGTGTCCCAGTGGTTAGAGACTACATAGTTACCATCACAGGGAATCAGAAGAAATCAAATCTTTCACTGAAACTGCATCCTCACCCTCAAAGCATGTTCAAGGACGCAAACATAAACGCAATTGAGCTCTTCAAAATGGACGACTCAACAGGTAATCTCGCTGGACCCAACCCAGACCCTCCTCTACAAGCCCCTGACCACAATGGTTCCCTTGAAAACTCAAAAAAGAAAAGCAGCGGCACCACAAGAACCCTTGCCGCCGTCGCAGGTGCAGTTTCCGGTGTCATTTTGCTCTCCTTTATTGTCGCTTCCTTCCTCGTCAAGCGCAAGAAGAACGCTTCCGTCCACAAGGGTTCCAAGCAAAACTACGGAACCTCTCGCGGCGGTGGTTCATCATCGCTGCCAACCAACCTCTGCCGCCACTTCTCAATCGCGGAAATCAGAGCCGCCACCAATAACTTCGACGAACTCTTCGTGGTGGGCGTGGGAGGCTTTGGCAACGTGTACAAAGGCTACATCGATGACGGTTCAACACCTGTTGCAATCAAAAGACTCAAACCGGGTTCTCAGCAAGGTGTACAGGAGTTCATGAACGAGATCGAAATGTTGTCTCAGCTTCGTCATCTTCATCTTGTTTCCCTCATCGGTTACTGCTACGAGAGTGACGAGATGATACTGGTTTACGATTTCATGGATCGCGGAACCCTTAGTGATCATCTCTATGACTCTGATAACTCGTCCTTGTCGTGGAAGCAAAGGCTGCAGATCTGCTTAGGTGCTGCACGTGGACTGCATTATCTGCATACAGGTGCGAAGCACATGATCATTCACCGTGACGTGAAGAGCACCAACATCTTATTGGATGAAAAATGGGTGGCCAAGGTTTCAGACTTCGGGTTATCCAGAATTGGGCCCACTGGTTCTTCAATGACCCATGTGAGCACTCTGGTGAAAGGTAGCATTGGATATTTAGACCCGGAGTATTACAAACGACAGCGTTTAACGGAGAAGTCTGACGTATACTCCTTTGGGGTGGTGCTCTTGGAGGTATTGTGTGGGAGGCAACCCTTGATCCGTACGGCGGAGAAGCAAAAGATGTCACTGGTGGATTGGGCAAAGCATCACTACGAGAAGGGCTTTCTTGGTGAGATTGTGGATCCCTCACTGAAGGGCCAGATAGCAGCTGAGTGTTTGCGCAAATTTGGTGAGGTTGCGTTGAGCTGTTTGCTTGAGGATGGGACTCAGAGACCCTCCATGAACGACATTGTTGGGATGTTGGAGTTTGTTCTGCAGCTTCAGGATAGTGCTGTTAATGGAGTGGTGCCTTTGTTGGTGAGTGGTGGGGATTGTGAAGATAGTGAGGACATGTTTAGCAGTACCCATAGTAGTATACAACTTTCGGATTATAGTAACAGCACTGGATTGAACACCTCAAGCTATGGGAGTAAGGAATCTGACAGGTTGATCCGGGAGAATGTTTTCTCTGAGATTAAGGATCCAAAGGGACGATAA

***GmCrRLK1L20*-RNAi fragment** **is** **as follows:**

CATGCCATGGTATAGACCCAGAGTATTATAAGCGGCATCATTTGACGGACAAGTCTGACGTGTACGCTTTCGGGGTAGTGTTGTTTGAGGTACTGTGCGCTCGTCCGCCTCTTATACGCAATGAAGATCCCAAACAGGAGTCGCTTGCTAAGTGGGTTAGGTACTGTTACCAAAGTGGGACCATGGACCAGATTGTGGACCCCACGTTGAAGGGGAGGATCGCGCCTGAATGCTTCCGGAGGTTTTGCCATATTGGGGTGAGTTGTTTGTCAGAGGTTGGGACGCAGAGGCCGTCGATGAAAGATGTTGTTTTCATGTTGGAGTCTACTCTGCAGGTGCAAGAGAGCGCGGAGAATGTAAAAAGAGGAAATTAGGATCCGATCGAAAAACGGGAGTCTGCCCCTAAGACAGATAAGCCGCCAAGAAGGCGCAAGTCAACCGCGAGTTGTTGTATCATATCTACTGACAAAGATCACAAATGGGATGGCTGATTAGATACCTTGGCCTCCCAGATCGATTCCTAATTTCCTCTTTTTACATTCTCCGCGCTCTCTTGCACCTGCAGAGTAGACTCCAACATGAAAACAACATCTTTCATCGACGGCCTCTGCGTCCCAACCTCTGACAAACAACTCACCCCAATATGGCAAAACCTCCGGAAGCATTCAGGCGCGATCCTCCCCTTCAACGTGGGGTCCACAATCTGGTCCATGGTCCCACTTTGGTAACAGTACCTAACCCACTTAGCAAGCGACTCCTGTTTGGGATCTTCATTGCGTATAAGAGGCGGACGAGCGCACAGTACCTCAAACAACACTACCCCGAAAGCGTACACGTCAGACTTGTCCGTCAAATGATGCCGCTTATAATACTCTGGGTCTATAGGTGACCC
