# Supplementary material for: Genome-Wide Analysis of the Catharanthus roseus RLK1-Like in Soybean and GmCrRLK1L20 Responds to Drought and Salt Stresses
Source: Front Plant Sci. 2021 Mar 18;12:614909. doi: 10.3389/fpls.2021.614909 (PMC8012678; doi:10.3389/fpls.2021.614909)
Supplement: Supplementary file 5 [file Table_4.docx]

The NCBI accession number of the five candidate soybean *GmCrRLK1L* genes

| **Number Gene ID Gene name NCBI** |  |
| --- | --- |
| 19 Glyma.13G053800 GmCrRLk1L19 LOC100799780  20 Glyma.13G054200 GmCrRLk1L20 LOC106795448  22 Glyma.13G054400 GmCrRLk1L22 LOC100499640  24 Glyma.15G042900 GmCrRLk1L24 LOC100305416  31 Glyma.18G270600 GmCrRLk1L31 LOC100305394 |  |
